# Complement C1q-dependent excitatory and inhibitory synapse elimination by astrocytes and microglia in Alzheimer’s disease mouse models

**DOI:** 10.1038/s43587-022-00281-1

**Published:** 2022-09-20

**Authors:** Borislav Dejanovic, Tiffany Wu, Ming-Chi Tsai, David Graykowski, Vineela D. Gandham, Christopher M. Rose, Corey E. Bakalarski, Hai Ngu, Yuanyuan Wang, Shristi Pandey, Mitchell G. Rezzonico, Brad A. Friedman, Rose Edmonds, Ann De Mazière, Raphael Rakosi-Schmidt, Tarjinder Singh, Judith Klumperman, Oded Foreman, Michael C. Chang, Luke Xie, Morgan Sheng, Jesse E. Hanson

**Affiliations:** 1 Stanley Center for Psychiatric Research, Broad Institute of MIT and Harvard, Cambridge, MA USA; 2 Department of Neuroscience, Genentech, South San Francisco, CA USA; 3 Department of Biomedical Imaging, Genentech, South San Francisco, CA USA; 4 Department of Microchemistry Proteomics and Lipidomics, Genentech, South San Francisco, CA USA; 5 Department of Pathology, Genentech, South San Francisco, CA USA; 6 Department of OMNI Bioinformatics, Genentech, South San Francisco, CA USA; 7 Department of Biomarker Development, Genentech, South San Francisco, CA USA; 8 Section Cell Biology, Center for Molecular Medicine, University Medical Center Utrecht, Utrecht University, Utrecht, The Netherlands

**Keywords:** Neuroimmunology, Alzheimer's disease, Glial biology, Ageing

## Abstract

Microglia and complement can mediate neurodegeneration in Alzheimer’s disease (AD). By integrative multi-omics analysis, here we show that astrocytic and microglial proteins are increased in Tau^P301S^ synapse fractions with age and in a C1q-dependent manner. In addition to microglia, we identified that astrocytes contribute substantially to synapse elimination in Tau^P301S^ hippocampi. Notably, we found relatively more excitatory synapse marker proteins in astrocytic lysosomes, whereas microglial lysosomes contained more inhibitory synapse material. C1q deletion reduced astrocyte–synapse association and decreased astrocytic and microglial synapses engulfment in Tau^P301S^ mice and rescued synapse density. Finally, in an AD mouse model that combines β-amyloid and Tau pathologies, deletion of the AD risk gene *Trem2* impaired microglial phagocytosis of synapses, whereas astrocytes engulfed more inhibitory synapses around plaques. Together, our data reveal that astrocytes contact and eliminate synapses in a C1q-dependent manner and thereby contribute to pathological synapse loss and that astrocytic phagocytosis can compensate for microglial dysfunction.

## Abstract (web summary)

In this multi-omics study, the authors identified C1q-dependent synapse elimination by both astrocytes and microglia in Alzheimer’s mouse models. While astrocytes preferentially removed excitatory synapses, microglia preferred inhibitory synapses.

## Main

Chronic neuroinflammation, manifested by gliosis and elevated levels of proinflammatory cytokines and synapse loss are hallmarks of AD^1,2^. One pathway that is aberrantly overactivated in mouse models and brains of patients with AD and drives neuronal damage and synapse loss is the classical complement pathway (CCP)^2–4^. CCP factors are abnormally elevated in brains and cerebrospinal fluid (CSF) of patients with AD^3–5^. Human genetic association studies support an involvement of the innate immune response including the complement pathway, in the pathogenesis of AD^2^. Components and regulators of the complement cascade are also genetically associated with schizophrenia and age-related macular degeneration^6–9^ and levels of various CCP molecules are increased in brains of patient and mouse models of AD, multiple sclerosis (MS), frontotemporal dementia and several other central nervous system (CNS) disorders^3,4,10–14^. This suggests that dysregulation of the complement pathway could play a role in diverse CNS disorders. In support of complement’s neurotoxic role, pharmacological or genetic inhibition of the complement pathway ameliorates neurodegeneration and synapse loss in mouse models of AD, MS, frontotemporal dementia and neuro-invasive virus infection^3,4,10–12,15^.

During development, microglia refine neuronal circuits by engulfing excess synapses^16^, which is at least in part CCP-dependent^17–19^. The CCP is initiated upon C1q binding to pathogens, apoptotic cells or other structures, including synapses that are destined for clearance. Subsequent activation of the CCP results in proteolytic cleavage of the complement factor C3, leading to microglial phagocytosis of complement-tagged synapses^17^. In addition to microglia, astrocytes have been shown to remove synapses during development, in the adult brain and in disease^20–22^. In contrast to microglia, however, synapse eating by astrocytes seems to be C1q-independent under physiological conditions^21^. While a synaptotoxic role for reactive astrocytes has been identified across different CNS diseases, including AD, Huntington’s disease, Parkinson’s disease and MS^22–24^, the molecular mechanisms remain largely unknown.

Here, we show that C1q deletion is neuroprotective in Tau^P301S^ transgenic mice (termed P301S hereafter), a mouse model of tauopathy and AD. Using multi-omics analysis and follow-up experiments, we unexpectedly found that astrocytes have a major role in the removal of excitatory synapses and also participate in the removal of inhibitory synapses in P301S mice in a C1q-dependent manner. In TauPS2APP mice, an AD mouse model that combines β-amyloid and Tau pathologies, we found that microglial phagocytosis of synapses near plaques is impaired in the absence of the AD risk gene *Trem2*. In TauPS2APP;Trem2^KO^ brains, astrocytes compensate for microglial dysfunction around plaques through increased eating of inhibitory synapses. Our data reveal an unexpected preference for excitatory versus inhibitory synapse engulfment by astrocytes versus microglia and support the idea that inhibition of complement is an attractive strategy to ameliorate neurodegeneration in AD.

## Results

### C1q deletion reduces neurodegeneration in P301S mice

**Fig. 1:**
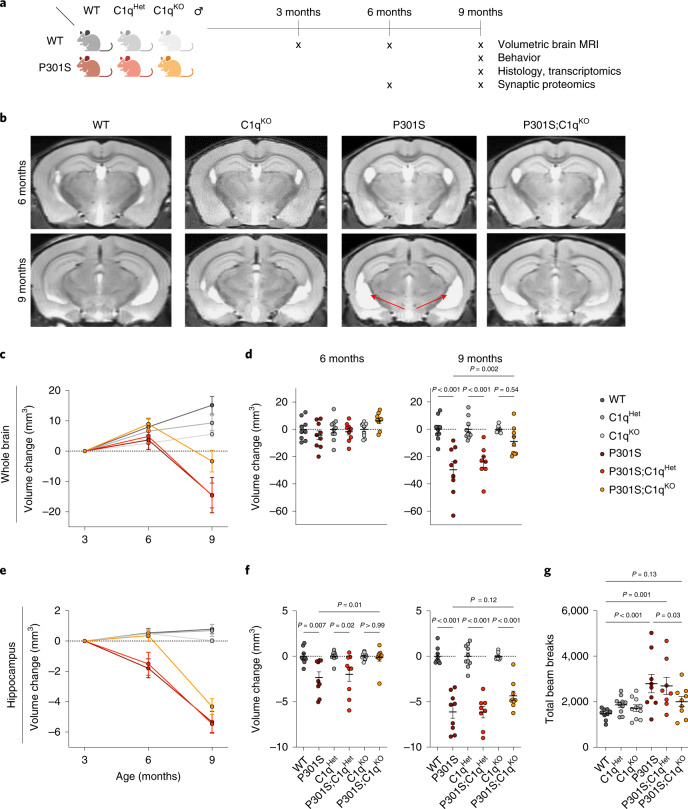
C1q deletion reduces neurodegeneration in P301S mice. **a**, Study design. Male P301S and C1q^KO^ mice were crossed as indicated and analyzed using longitudinal volumetric brain MRI, behavioral hyperactivity and pathological analysis, transcriptomics and synapse proteomics. **b**, Representative volumetric MRI images in male mice at 6 and 9 months of age. Arrows indicate hippocampal atrophy and ventricle enlargement in P301S mice. **c**, Longitudinal volumetric MRI quantification of whole brain volume changes in indicated mouse genotypes at 6 and 9 months (normalized to 3 months). **d**, Whole brain volume changes in indicated mouse genotypes at 6 and 9 months of age. P301S transgenic mice were normalized to non-transgenic mice with the same C1q genotype for comparison. **e**, Longitudinal volumetric MRI quantification of hippocampal brain volume changes in indicated mouse genotypes at 6 and 9 months (normalized to 3 months). **f**, Hippocampus volume changes in indicated mouse genotypes at 6 and 9 months of age. P301S transgenic mice were normalized to non-transgenic mice with the same C1q genotype for comparison. **g**, Nine-month-old mice were evaluated in the open field behavioral test by measuring total beam breaks to assess for behavioral hyperactivity. Each dot represents the values from one mouse. *n* = 8–10 mice per genotype (**c**–**g**). One-way ANOVA with Tukey’s multi-comparisons test (**d**,**f**) and one-way ANOVA with Fisher’s least significant difference test (**g**). All data are presented as mean ± s.e.m. Source data

To investigate the role of C1q in the progressive neurodegeneration of P301S mice^4^, we genetically ablated C1q and analyzed males using volumetric brain magnetic resonance imaging (MRI), behavioral and pathological analysis, as well as transcriptomics and synapse proteomics (Fig. 1a).

**Extended Data Fig. 1:**
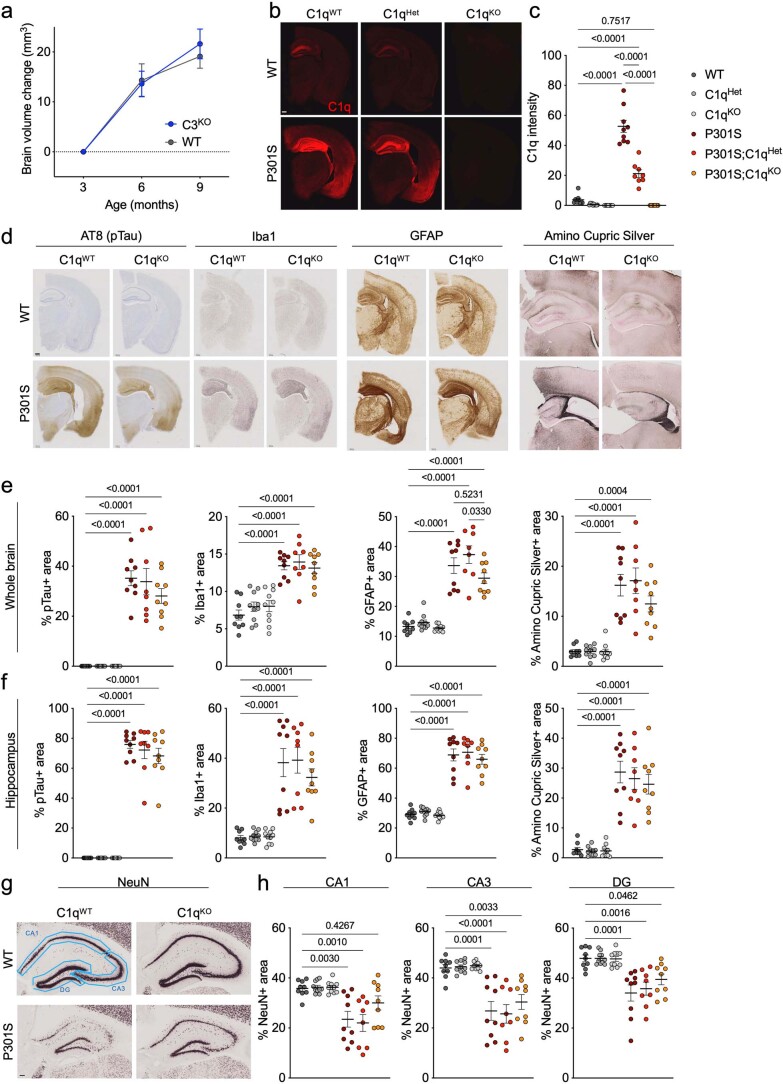
Immunohistochemical characterization of C1q experimental cohort. (**a**) Longitudinal volumetric T2 weighted MRI quantification of whole brain volume changes in WT and C3^KO^ mice at 6 and 9 months (normalized to 3 months). (**b**) Example C1q immunofluorescence images from hemibrains of each genotype used in the study. (**c**) Quantification of C1q immunofluorescence in the whole brain of 9-month-old mice. (**d**) Representative images showing AT8 (pTau), Iba1, GFAP in hemibrains and Amino Cupric Silver staining in the hippocampus. (**e**) Quantification of pTau, Iba1, GFAP and Amino cupric silver positive area in whole brains. (**f**) Quantification of the same markers as in E) with analysis restricted to the hippocampus. (**g**) Representative images showing NeuN staining in each genotype with example ROIs for the CA1, CA3, and dentate gyrus (DG), subfields are illustrated on the first image. (**h**) Percentage of NeuN+ area in hippocampal CA1, CA3 and DG subregion across genotypes. Each dot shows average data from one mouse. 13–14 mice/genotype were used for volumetric MRI experiment in a), 8–10 mice/genotype were analyzed by immunohistochemistry in c-h). One-way ANOVA with Tukey multiple comparison test was used. All data are presented as mean ± SEM. Source data

We monitored brain volume longitudinally at 3, 6 and 9 months of age. Compared to wild-type (WT) mice, the rate of brain volume growth during maturation in C1q-deficient mice was reduced in a gene dose-dependent manner (Fig. 1b,c; WT versus C1q^KO^; *P* = 0.04; two-way analysis of variance (ANOVA) with Dunnett’s test). We did not observe differences in brain volume changes in C3^KO^ mice (Extended Data Fig. 1a), suggesting that C1q might have CCP-independent physiological functions in the brain. In P301S mice, there was a marked decrease in brain volume between 6 and 9 months, reflecting neurodegeneration (Fig. 1c,d). In P301S;C1q^KO^ mice, brain volume loss was less severe and brain volume was not significantly different from C1q^KO^ brains at 9 months (Fig. 1c,d)^4^. In the hippocampus, which is the brain region most severely affected by Tau pathology and gliosis^3^, we observed a reduction in volume even at 6 months and further atrophy between 6 and 9 months in P301S mice (Fig. 1e,f). In P301S;C1q^KO^ mice hippocampal volume loss was delayed, with significant protection at 6 months (Fig. 1e,f). In contrast to the protection afforded by homozygous C1q knockout (KO), P301S;C1q^Het^ mice resembled P301S mice, implying that greater than 50% reduction of C1q is needed for protection against Tau^P301S^ neurodegeneration.

To test for behavioral consequences of C1q deletion, we measured locomotor activity in an open field in 9-month-old mice (Fig. 1g). As expected, P301S mice exhibited hyperactivity, which is thought to be caused by hippocampal damage^3^. While there was no effect of C1q genotype on locomotor activity in the absence of the P301S transgene, hyperactivity was rescued in P301S;C1q^KO^, but not P301S;C1q^Het^ mice (Fig. 1g).

**Extended Data Fig. 2:**
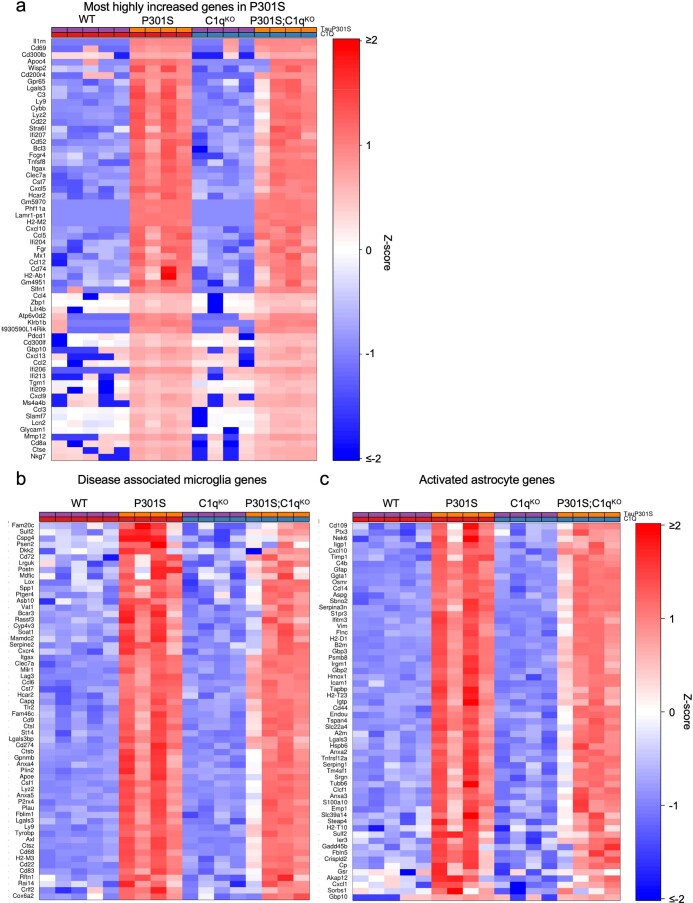
C1q deletion does not impact transcriptional changes in P301S mice. (**a**) Heatmap showing Z score of genes that were most highly up-regulated in P301S vs. WT mice (without respect to C1q genotype). No genes showed DE when comparison was made between P301S and P301S;C1qKO mice (adjusted p <0.05) except for *C1qc* (log2FC = −6.44, p = 0.000256). (**b**) Heatmap showing Z score of top 60 DAM genes taken from the list in^67^. (**c**) Heatmap showing Z score of top astrocytes activated genes taken from the list in^4^.

We then analyzed brain histopathology in 9-month-old mice. C1q immunoreactivity was strongly increased in P301S brains, compared to a ~50% reduction in P301S;C1q^Het^ brains and was undetectable in P301S;C1q^KO^ brains (Extended Data Fig. 1b,c). P301S brains were characterized by strong phospho-Tau immunoreactivity, microgliosis and astrogliosis measured by increased Iba1^+^ and glial fibrillary acidic protein (GFAP)^+^ area, respectively^3^ (Extended Data Fig. 1d–f). There was no difference in these histopathologic readouts in P301S;C1q^Het^ versus P301S mice and a slight trend toward reduction in P301S;C1q^KO^ compared to P301S mice (Extended Data Fig. 1d–f). We observed trends toward reduced amino cupric staining, which reflects damaged neurons and increased density of the neuronal marker NeuN in P301S;C1q^KO^ hippocampi (Extended Data Fig. 1e–h). Bulk RNA-seq of P301S hippocampi showed upregulation of many genes, including multiple markers of activated microglia and astrocytes (Extended Data Fig. 2); however, C1q deletion had no effect on these major transcriptomic changes in P301S hippocampi (Extended Data Fig. 2). Together, these results show that C1q deletion reduces P301S brain degeneration and normalizes behavior without having a significant effect on the extent of Tau pathology, gliosis or glial transcriptional changes. Thus, the protective effect of C1q deletion seems to act downstream of tauopathy and the overall glial response.

### C1q^KO^ blunts proteomic changes in P301S synapses

**Fig. 2:**
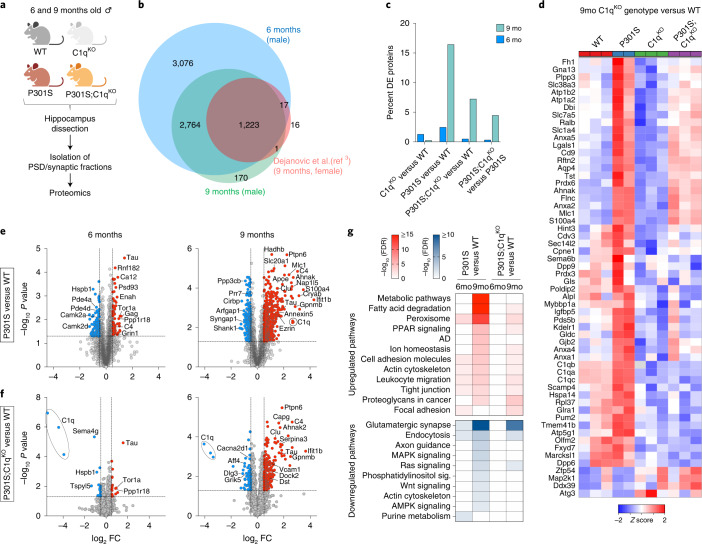
C1q deletion blunts proteomic changes in P301S synapses. **a**, Experimental design of hippocampal PSD proteome analysis. Hippocampi from 6- and 9-month-old male mice were dissected and isolated synapse fractions were analyzed by TMT multiplex proteomics (Methods). **b**, Venn diagram showing the number of identified proteins and overlap between synapse proteomes in the 6- and 9-month cohort from this study and synapse proteome from 9-month-old female mice described previously^3^. **c**, Percentage of DE proteins in the indicated genotype comparisons at 6 and 9 months. **d**, A heat map showing *z* scores across genotypes for proteins that were DE between C1qKO and WT mice (regardless of P301S genotype) (*P* ≤ 0.05; FC ≥ 5). **e**,**f**, Volcano plots showing the comparison between P301S versus WT and P301S;C1q^KO^ versus WT synapse proteomes at 6 and 9 months. MSstats was used to calculate log_2_FC and standard error utilizing a linear mixed-effects model that considered quantification from each peptide and biological replicate per protein. *P* values were then calculated by comparing the model-based test statistic to a two-sided Student’s *t*-test distribution. Significantly up- and downregulated proteins (*P* < 0.05, log_2_FC ± 0.5) are shown in blue and red circles, respectively. Selected DE proteins are labeled with their protein or gene name. **g**, Selected up- or downregulated KEGG pathways in P301S versus WT and P301S,C1q^KO^ versus WT synapse proteomes at 6 and 9 months. Only DE proteins were included for pathway analysis. Source data

We next examined changes in synaptic protein composition across P301S and C1q genotypes. We isolated hippocampal postsynaptic density (PSD) fractions from 6- and 9-month-old male mice to detect changes at an early and an advanced stage of disease, respectively (Fig. 2a). These fractions are highly enriched in proteins of the PSD and postsynaptic membrane; they also contain components of the presynaptic terminal, transsynaptic adhesion molecules and some glia-specific proteins (that might reflect close interactions of glial cells with synapses)^3,25,26^. Thus, we use the terms PSD and synapse fraction interchangeably throughout the study. Multiplexed tandem mass tag (TMT) proteomics detected a total of 7,101 proteins in PSD fractions from 6-month-old mice and 4,175 proteins in those from 9-month-old mice (Fig. 2b and Supplementary Table 1). While our previous analysis of 9-month-old P301S females using label-free proteomics detected fewer proteins^3^, nearly all of them were also found in the current study (Fig. 2b).

**Extended Data Fig. 3:**
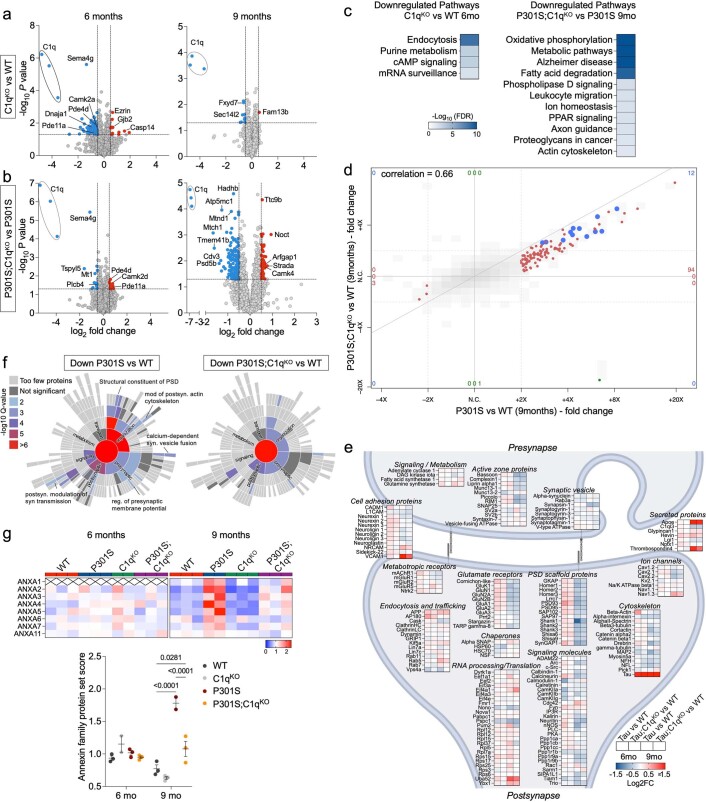
C1q-dependent synapse proteome changes in P301S mice. (**a,b**) Volcano plots showing the comparison between A) C1q^KO^ vs WT and B) P301S;C1q^KO^ vs P301S synapse fraction proteomes at 6 and 9 months. MSStats was used to calculate log2(fold change) and standard error utilizing a linear mixed-effects model that considered quantification from each peptide and biological replicate per protein. P values were then calculated by comparing the model-based test statistic to a two-sided Student t-test distribution. Significantly up- and downregulated proteins (p-value < 0.05, log2FC ± 0.5) are shown in blue and red circles, respectively. Selected differentially expressed proteins are labeled with their protein or gene name. (**c**) KEGG pathways significantly downregulated in synapses from 6 months old C1q^KO^ mice and 9 months old P301S;C1q^KO^ vs P301S mice. (**d**) Scatterplot comparison of PSD proteomes from 9 months old P301S vs WT mice (x-axis) and P301S;C1q^KO^ vs WT mice (y-axis). Note that protein changes in this comparison are larger in P301S compared to P301S;C1q^KO^ synapse fractions, suggesting that C1q deletion blunts changes in P301S mice. Red indicates significantly different (p-value <0.05) in P301S vs. WT, but not significantly different in P301S;C1q^KO^ vs WT (97 proteins), green indicates significantly different in P301S;C1q^KO^ vs WT, but not significantly different in P301S vs. WT (only C1qC), and blue indicates significantly different in both comparisons (12 proteins). P-values and fold changes were calculated by MSStats, as described in methods. (**e**) Schematic representation of an excitatory synapse with the localization of selected proteins grouped by their function. Heatmaps show protein log2 fold-changes for individual genotype and age comparison. (**f**) SynGO analysis of downregulated proteins in P301S vs WT and P301S;C1q^KO^ vs WT synapse proteomes at 9 months. (**g**) Heatmaps of normalized protein expression of annexins across genotypes in synapses at 6 and 9 months. The annexin protein set score is shown below. In g) each dot shows data from one mouse. 2–3 mice/genotype were used. Two-way ANOVA with Šidak’s test. All data are presented as mean ± SEM. Source data

Synapse fractions from C1q^KO^ mice showed only a small number of differentially expressed (DE) proteins (defined as log_2_fold change (FC) ± 0.5, nominal *P* < 0.05) compared to WT at both ages (Fig. 2c and Extended Data Fig. 3a,c). In 6-month-old P301S synapse fractions we found 108 downregulated and 68 upregulated proteins (2.5% of total proteins; Fig. 2c,e). At 9 months, there were 253 downregulated and 434 upregulated proteins, corresponding to 16.5% of total proteins (Fig. 2c,e). By contrast, P301S;C1q^KO^ synapse fractions showed only 17 decreased and 19 increased DE proteins (0.5% of total proteins) at 6 months and 79 decreased and 224 increased DE proteins (7% of total proteins) at 9 months (Fig. 2c,f). Consistently, we found many DE proteins when comparing P301S;C1q^KO^ to P301S synapses at 9 months (Fig. 2c and Extended Data Fig. 3b) and reductions in Tau-dependent changes with C1q deletion (Extended Data Fig. 3d). C1q-deficiency did not affect Tau levels in synapse fractions of P301S brains (Fig. 2e,f). Thus, C1q deletion blunted age-dependent changes induced by Tau pathology even though C1q deletion had little effect in non-transgenic mice (Fig. 2d and Extended Data Fig. 3d). As C1q deletion did not significantly alter the transcriptomic changes in P301S brains (Extended Data Fig. 2), synapse proteome changes are likely driven by local protein changes at the synapse.

We assessed the impact of the P301S transgene and C1q deletion on various functional classes of proteins in the synapse proteome (Extended Data Fig. 3e). Many core PSD proteins, such as glutamate receptors, scaffolding proteins, synaptic adhesion molecules and certain presynaptic active zone proteins tended to be increased in P301S compared to WT synapses at 6 months but reduced at 9 months (Extended Data Fig. 3e). This could reflect compensatory synaptic changes at early disease stage that are overcome by synaptic damage at the later stage. Using the synaptic Gene Ontology tool SynGO^27^, we confirmed that the decreased DE proteins in 9-month-old P301S PSDs were significantly enriched with synapse organization and canonical pre- and postsynaptic proteins (Extended Data Fig. 3f). While qualitatively similar synaptic functions were affected in P301S;C1q^KO^ synapse fractions, the changes were less significant than in P301S PSDs (Extended Data Fig. 3f).

KEGG pathway analysis of the DE proteins in P301S synapses showed significant enrichment in ‘metabolic pathways’ (containing mainly mitochondrial proteins such as Echs1, Maob and Atp8), ‘fatty acid degradation’ (for example Adh5, Hadha and Hadh), ‘peroxisome’ (for example Dhrs4, Ehhadh and Abcd1), ‘peroxisome proliferator-activated receptors (PPAR)’ signaling (for example Cpt1a and Cpt2), ‘Alzheimer’s disease’ (for example Adam10 and APOE) and ion homeostasis (for example Slc4a4 and Aqp4), especially prominent at 9 months (Fig. 2g). Increases in these pathways were relatively subdued in P301S;C1q^KO^ synapses, with no significantly increased pathways at 6 months and changes at 9 months resembling alterations seen in 6-month-old P301S PSDs (Fig. 2g). Consistently, most pathways that were increased in 9-month-old P301S synapses, were decreased in the P301S;C1q^KO^ versus P301S comparison (Extended Data Fig. 3b,c). Notably, ‘metabolic pathways’, the most significantly increased pathway in 9-month-old P301S synapses, was not significantly induced in P301S;C1q^KO^ samples (Fig. 2g). We also noted that annexins were among the most highly induced proteins in 9-month-old TauP301S synapses and were partly normalized in P301S;C1q^KO^ synapses (Fig. 2d and Extended Data Fig. 3g).

Analysis of decreased DE proteins in P301S synapses highlighted pathways including ‘glutamatergic synapse’ (for example Shank1 and Grin2a), ‘endocytosis’ (for example Vps4a and Rab11Fip2), ‘axon guidance’ (for example Ntng1 and Smad2), ‘MAPK-’ (for example Mapk1 and Map4k4), ‘Ras-’ (for example Ksr1 and Syngap1), ‘phosphatidylinositol-’ (for example Dgkb and Dgkq) and ‘Wnt-signaling’ (Apc2 and Dvl3) and ‘AMPK-signaling’ (Ppp2r5c and Prkaa2) (Fig. 2g). No pathway was significantly decreased in 6-month-old P301S;C1q^KO^ synapses and only ‘glutamatergic synapse’, ‘endocytosis’ and ‘Ras signaling’ were significantly decreased at 9 months (Fig. 2g). Different proteins of the ‘actin cytoskeleton’ pathway were significantly increased (for example ezrin and gelsolin) or decreased (for example Baiap2 and Pak6) in P301S PSD fractions. At least some of the increased actin-regulating proteins in the synapse fractions are expressed by glial cells that presumably associate with synapses (see below).

**Extended Data Fig. 4:**
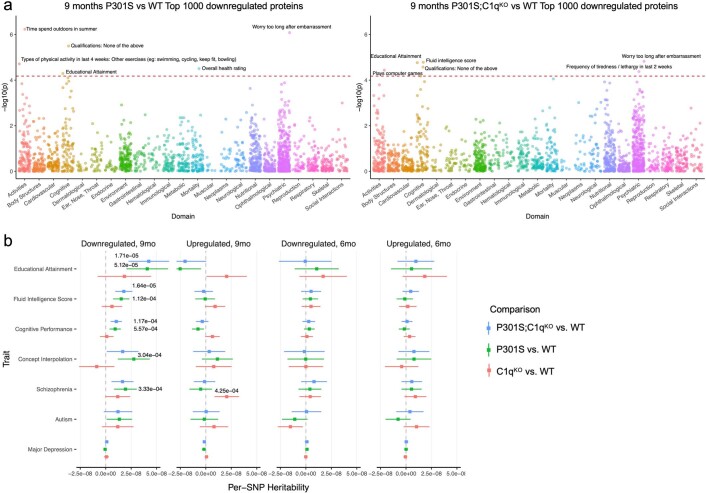
Heritability enrichment analyses in differentially abundant proteins identified in P301S and P301S;C1q^KO^ synapse fractions. (**a**) Manhattan plots of per-SNP enrichment p-values for the top 1000 down-regulated proteins in 9-month P301S and P301S;C1q^KO^ PSDs for 752 traits across 23 of the 24 UK Biobank Domains. The dotted red line corresponds to the Bonferroni threshold at 0.05 correcting for 752 traits (6.65 ×10^−5^). (**b**) Per-SNP heritability coefficients and 95% confidence intervals of seven select cognitive and psychiatric traits for the top 1000 up- or down-regulated proteins in C1qKO, P301S and P301S;C1q^KO^ PSDs at 6 and 9 months. The GWAS for the seven cognitive and psychiatric traits have sample sizes between 46,350 to 1.1 million individuals (see methods). Dots with p-value < 0.001 were labeled in the graph. All *P* values are one-sided and calculated using s-LDSC.

As mutations in synaptic proteins cause a variety of neurological and neuropsychiatric diseases, we investigated whether DE proteins were enriched for genetic signals in genome-wide association studies (GWAS) of relevant traits and disorders^28–34^. For the 1,000 most up- and downregulated proteins in P301S versus WT synapses, we tested for polygenic signal in 752 traits primarily from the UK Biobank and selected GWAS studies using stratified linkage disequilibrium (LD)-score regression (Methods). After grouping traits into 23 categories or domains, we found that the downregulated proteins in 9-month-old P301S and P301S;C1q^KO^ synapses had significant enrichments in cognitive, psychiatric and activities domains, which included educational attainment, fluid intelligence score, cognitive performance and concept interpolation (Extended Data Fig. 4a). In contrast there was limited enrichment for these traits in the upregulated proteins in 9-month-old P301S PSD fractions or in DE proteins at 6 months (Extended Data Fig. 4b). This suggests that proteins downregulated in 9-month-old P301S and P301S;C1q^KO^ synapses have relevance in human cognitive function and behavior.

### C1q-dependent elevation of glial proteins at P301S synapses

**Fig. 3:**
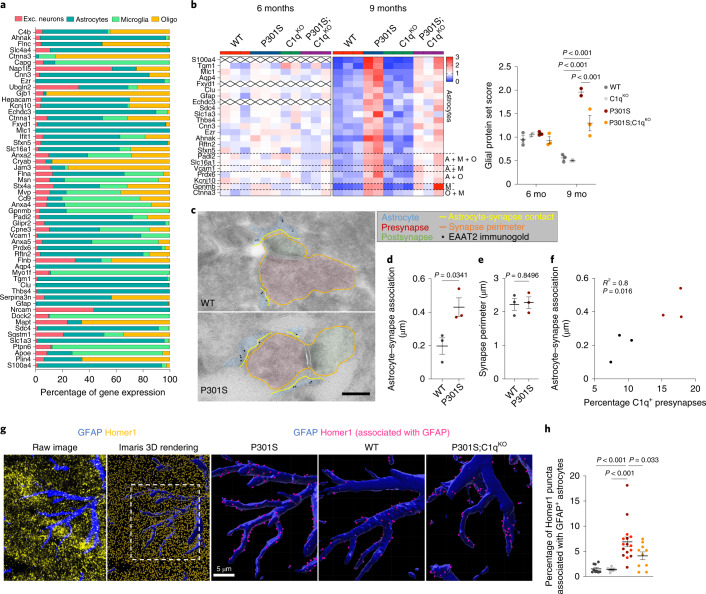
Glial proteins are elevated at P301S synapses and normalized by C1q^KO^. **a**, Cell-type-specific expression of genes that encode the most highly increased proteins in P301S synapses at 9 months. Percentage of gene expression in the major brain cell types (excitatory (exc.) neurons, astrocytes, microglia and oligodendrocytes (oligo)) was calculated based on pseudobulk analysis of scRNA-seq data from P301S mice. **b**, Heat maps showing z scores for normalized levels of glial proteins across genotypes in synapses at 6 and 9 months. Genes from **a** were defined as glial if the percentage of gene expression in excitatory neuron was <4%. Dotted lines indicate the cell type(s) that mainly express the corresponding gene. A, astrocyte; M, microglia; O, oligodendrocyte. Glia protein set score (right). **c**, Representative immunoEM images of EAAT2 in DG. Presynapses are pseudo-colored in red, postsynapses in green. EAAT2^+^ astrocyte processes are shown in blue. The synapse perimeter is outlined in orange and the astrocytic plasma membrane that is in contact with the synapse is in yellow. Scale bar, 200 nm. **d**, Length of astrocyte plasma membrane in association with the synapse in WT and P301S mice. **e**, Quantification of synapse perimeter in WT and P301S mice. **f**, Two-tailed Pearson’s correlation of astrocyte–synapse association and percentage of C1q-labeled presynapses, which was quantified previously in the same mice^3^. **g**, Representative images showing the raw confocal immunofluorescence and the corresponding Imaris-processed image of GFAP (blue) and Homer1 (yellow) from a P301S brain. Inset shows three-dimensional (3D)-reconstructed GFAP^+^ astrocyte processes in the P301S brains and representative images from WT and P301S;C1q^KO^ brains. Only Homer1 puncta that associate with astrocytes are shown (pink dots). **h**, Fraction of Homer1 puncta associated with astrocytes. Data were analyzed by two-way ANOVA with Tukey’s multi-comparisons test (**b**); two-tailed unpaired Student’s *t*-test (**d**,**e**) (10–16 astrocyte-synapses were quantified per mouse) and one-way ANOVA with Dunnett’s multiple comparisons test (**h**). Each dot shows average data from one mouse; *n* = 2–3 mice per genotype (**b**); *n* = 3 mice per genotype (**d**,**e**) and *n* = 7–10 mice per genotype (**h**). All data are presented as mean ± s.e.m. Source data

**Extended Data Fig. 5:**
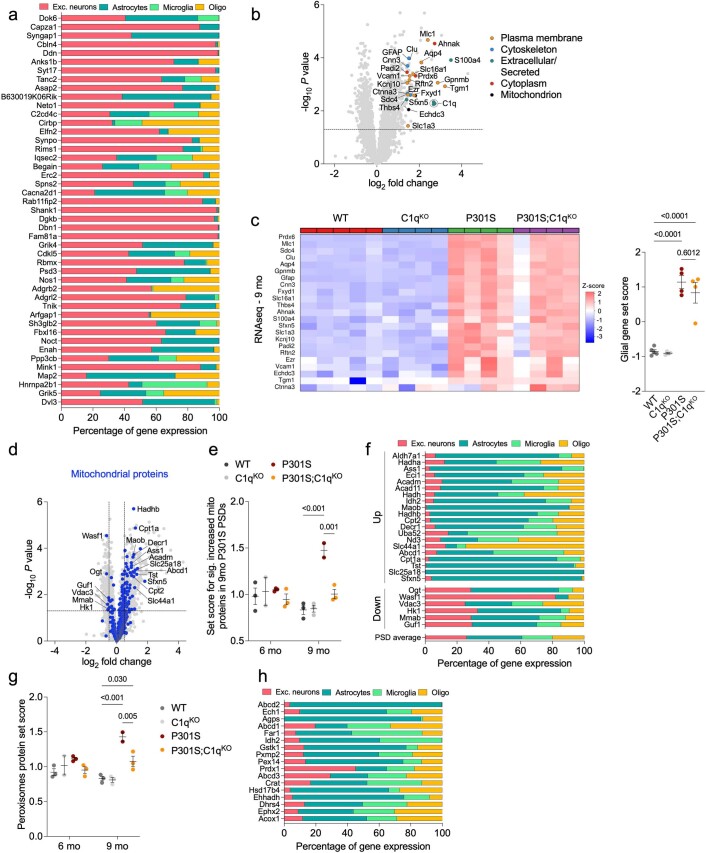
Abundance of glial proteins at synapses and expression of their corresponding genes. (**a**) Cell-type-specific expression of genes that encode the most highly decreased proteins in P301S synapses at 9 months. Percentage of gene expression was calculated based on pseudobulk analysis of scRNAseq data from P301S mice. (**b**) Volcano plot comparing P301S and WT synapse proteomes highlighting increased proteins that are selectively expressed by glial cells (<5% gene expression by neurons) and their annotated subcellular localization. MSStats was used to calculate log2(fold change) and standard error utilizing a linear mixed-effects model that considered quantification from each peptide and biological replicate per protein. P values were then calculated by comparing the model-based test statistic to a two-sided Student t-test distribution. (**c**) Heatmap showing z-scores from bulk RNAseq across genotypes for astrocyte and microglia specific genes encoding proteins in Fig 3c. The glial gene set score is shown on the right. (**d**) Mitochondrial proteins (blue dots) highlighted in volcano plots comparing 9 months old P301S vs WT synapse proteomes. Statistical tests were done as in panel B. The most highly up- or downregulated mitochondrial proteins are labeled using their gene or protein name. (**e**) Gene set score for mitochondrial proteins that are significantly increased in P301S synapses are shown across genotypes and age as indicated. (**f**) Cell-type-specific expression of genes encoding mitochondrial proteins that are up- or down-regulated in P301S PSDs at 9 months. Percentage of gene expression was calculated based on pseudobulk analysis of scRNAseq data from P301S mice. (**g**) Gene set score for peroxisome proteins that are significantly increased in P301S synapses are shown across genotypes and age as indicated. (**h**) Cell-type-specific expression of genes encoding peroxisome proteins that are up- or down-regulated in P301S PSDs at 9 months. Each dot shows data from one mouse. 2–5 mice/genotype were used. In c one-way ANOVA with Tukey multiple comparison test and in e, g two-way ANOVA with Tukey multiple comparison test was used. Percentage of gene expression in a, f and h is based on scRNAseq data from P301S mice. Source data

We noticed that a number of canonical astrocyte-specific proteins, such as Aqp4, Mlc1 and Slc1a4 were increased in 9-month-old P301S synapse fractions in a C1q-dependent manner (Fig. 2d). While contamination with astrocyte proteins is a possibility, we also considered whether the copurification of astrocytic proteins with synaptic preparations might result from the close interaction of astrocyte processes with synapses^25^. We generated pseudobulk single-cell RNA-sequencing (scRNA-seq) data from P301S hippocampi and found that the majority of the 55 most highly upregulated proteins were predominantly expressed by glial cells, rather than by excitatory neurons (Fig. 3a). In contrast, the most highly decreased proteins were mainly produced by excitatory neurons (Extended Data Fig. 5a). Besides Aqp4 and Mlc1, many other upregulated DE proteins were selectively or predominantly expressed by astrocytes (for example, clusterin, Slc1a3, Sdc4, AHNAK, ezrin, GFAP and Thbs4) (Fig. 3a). A smaller number of upregulated proteins were expressed predominantly by microglia, (for example, Gpnmb and Myo1f) (Fig. 3a). We hypothesized that the surge in glial proteins in 9-month-old P301S synapse fractions might reflect an increase in their secretion and subsequent accumulation at synapses and/or an increase in contact of glial processes with damaged synapses. Consistently, many of the increased glial proteins are either localized at the plasma membrane (for example, Slc16a1 and Aqp4), cytoskeleton (for example, ezrin) or are extracellular/secreted (clusterin and Thbs4; Extended Data Fig. 5b) and are known to be present in astrocyte processes^35,36^. Notably, the increase in glial proteins in P301S synaptic fractions was C1q-dependent (Fig. 3b). This argues against an indiscriminate contamination of PSD preps with glial-derived proteins. The relative reduction in glial proteins in P301S;C1q^KO^ synapses seems to be due to specific changes in association of glial proteins with synaptic fractions, rather than overall abundance of glial proteins, as the expression of the corresponding genes was not significantly different in P301S versus P301S;C1q^KO^ brains (Extended Data Fig. 5c).

Among the most highly increased proteins in P301S synapse fractions were astrocyte-specific mitochondrial proteins Echdc3 and Sfxn5 (Fig. 3a,b). Many mitochondrial proteins were increased in P301S synapse fractions in an age- and C1q-dependent manner (Extended Data Fig. 5d,e). Energy metabolism differs between CNS cell types^37^ and ‘metabolic pathways’ were strongly elevated in P301S but not P301S;C1q^KO^ synapses at 9 months (Fig. 2g). Although mitochondria can be transferred from astrocytes to neurons under pathological conditions^38^, we reasoned that mitochondria in synaptic fractions might originate from glial processes that were in close physical contact with synapses. Consistently, the 20 most highly increased mitochondrial proteins in P301S synapses are predominantly expressed by astrocytes (for example, Maob, Cpt1a and Tst, Slc25a18), whereas significantly decreased mitochondrial proteins had a broad expression pattern, including stronger neuronal production (for example, Wasf1) (Extended Data Fig. 5f). Similarly, the peroxisomal proteins that were increased in 9-month-old P301S synapse fractions in a C1q-dependent manner (Extended Data Fig. 5g) were also predominantly expressed by astrocytes (Extended Data Fig. 5h).

**Extended Data Fig. 6:**
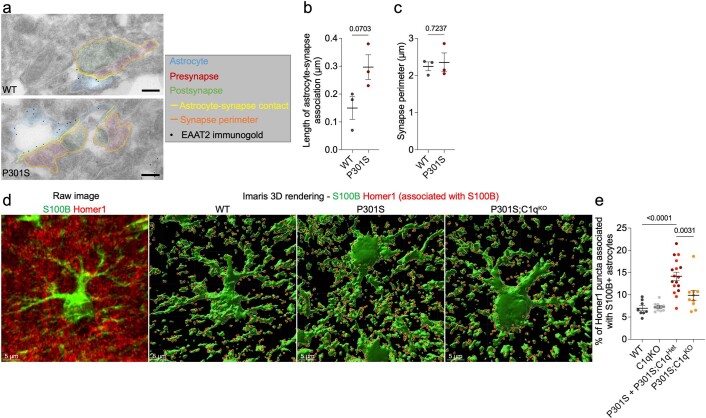
IEM and IHC analysis of astrocyte–synapse interaction. (**a**) Representative immunoEM images of EAAT2 in hippocampal CA1 region. Presynapses are pseudo-colored in red, postsynapses in green. EAAT2^+^ astrocyte processes are shown in blue. The synapse perimeter is outlined in orange and the astrocytic plasma membrane that is in contact with the synapse in yellow. Scale bar = 200 nm. (**b**) Length of the astrocytic plasma membrane associated with the synapse in CA1 region from WT and P301S mice. (**c**) Quantification of synapse perimeter in CA1 region from WT and P301S mice. (**d**) Representative confocal image and Imaris 3D reconstructions of immunostained S100B (green) and Homer1 (red). In the 3D reconstructions only S100B-associated Homer1 puncta are shown. Scale bar = 5µm. (**e**) Percentage of Homer1 puncta that associated with S100B^+^ astrocytes. B, c Unpaired two-tailed t-test; e One-way ANOVA with Dunnett multi comparison test. Each dot shows average data from one mouse, in b,c) n = 3 mice/genotype, in e) n = 8–10 mice/genotype. All data are presented as mean ± SEM. Source data

We next used immuno-electron microscopy (IEM) to determine whether there was altered physical association of astrocytes with synapses in P301S mice. We identified astrocyte processes by immunolabeling for the astrocyte-specific glutamate transporter EAAT2/Glt1 and quantified the length of astrocyte processes that are in contact with synapses in the hippocampus dentate gyrus (DG) and CA1 region (Fig. 3c and Extended Data Fig. 6a). Compared to WT mice, the length of astrocytic processes that associated with synapses was increased ~twofold in the DG and CA1 region in P301S mice (Fig. 3d and Extended Data Fig. 6b). The average synaptic perimeter was unchanged in P301S mice, implying that astrocytes contact a larger fraction of synaptic membrane (Fig. 3e and Extended Data Fig. 6c). Notably, the extent of astrocyte–synapse association correlated significantly with the percentage of C1q-labeled presynapses^3^ (Fig. 3f).

As an orthogonal measurement of astrocyte–synapse interaction across all genotypes, we quantified the spatial contact of excitatory synapses (Homer1 puncta) with the surface of GFAP^+^ astrocytes in the hippocampal CA1 region using confocal microscopy (Fig. 3g). As loss of one copy of C1q had no impact on pathology in P301S mice (Fig. 1 and Extended Data Fig. 1), we grouped P301S and P301S;C1q^Het^ brains in this analysis to increase statistical power. While there was no difference between C1q^KO^ versus WT hippocampi, we observed a significant increase in surface GFAP–Homer1 association in P301S hippocampi, which was significantly reduced in P301S;C1q^KO^ versus P301S hippocampi (Fig. 3h). Using the cytoplasmic astrocyte marker protein S100b to render astrocyte volume confirmed the C1q-dependent increase in astrocyte–Homer1 association in P301S mice (Extended Data Fig. 6d,e). Together, our analysis of synapse proteomics data, IEM and immunohistochemistry (IHC) measurements suggests that at a stage of disease with synapse engulfment and loss, astrocytes can increase their physical interaction with synapses in a C1q-dependent manner.

### Glial proteins are elevated in synapses of Alzheimer’s brain

**Fig. 4:**
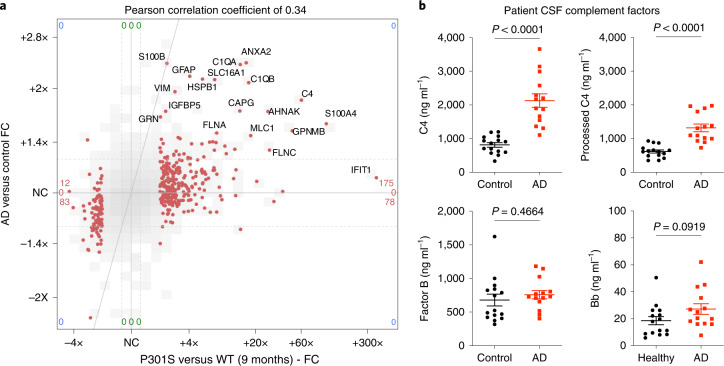
Glial proteins are increased in human AD synapse fractions and C4 is elevated in AD CSF. **a**, Scatter-plot comparison of synapse proteomes from 9 months old P301S versus WT mice (*x* axis) and AD versus control patients (*y* axis)^39^. Only orthologous protein pairs that were present in both datasets are shown. Of the 315 proteins that were significantly increased in P301S versus WT mice (*P* < 0.05, FC > 2) regardless of C1q genotype), 175 were increased in patients with AD versus controls, including the labeled proteins. Overall correlation of 0.34. NC, no change. **b**, Levels of total and processed C4 and Factor B and processed Bb fragment in CSF from controls and patients wth AD. Each dot represents the values from one individual. CSF samples from 15 controls and 14 patients with AD were analyzed (the same patients identified in previous works^4^; cohort 1). Data were analyzed by two-tailed unpaired Student’s *t*-test. All data are presented as mean ± s.e.m. Source data

We wondered whether glial proteins are also elevated in synaptic fractions from patients with AD. Comparison with a recently published synaptoneurosome proteome from the superior temporal gyrus (BA 41/42) in patients with AD^39^ revealed a notable positive correlation between changes in human AD versus control and 9-month-old P301S versus WT mice synapse proteomes (Fig. 4a). Notably, glial proteins that were elevated in P301S synapse fractions, including complement factors C1q and C4, astrocytic marker proteins MLC1 and GFAP, microglial GPNMB and AHNAK and annexins were among the most highly increased proteins in AD synaptoneurosomes (Fig. 4a).

**Extended Data Fig. 7:**
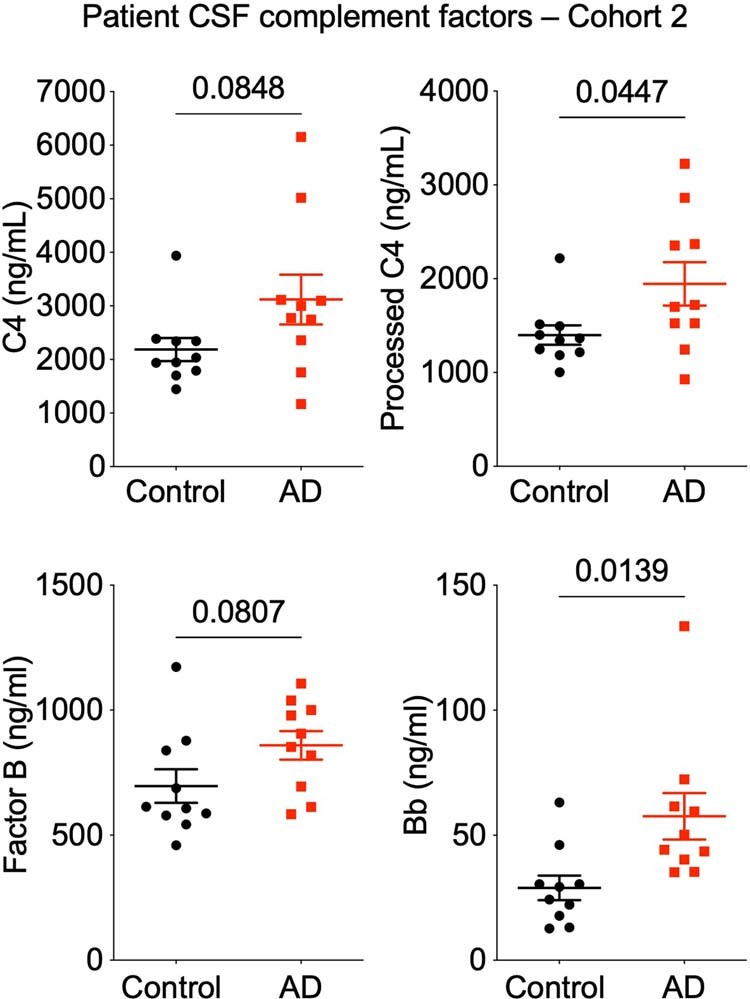
Complement C4 and Factor B concentrations in AD CSF. Levels of total and processed C4 and Factor B and processed Bb fragment in CSF from controls and AD patients. Each dot represents the values from one individual. CSF samples from 10 controls and 10 AD patients were analyzed (the same patients from^4^, cohort 2). Unpaired two-tailed t-test. All data are presented as mean ± SEM. Source data

We reasoned that elevated levels of C4 and activation of the CCP could be detectable in CSF from patients^4^, which might be a useful biomarker of complement activation. Indeed, total and processed (cleaved and activated), C4 concentrations were significantly increased in CSF from patients with AD (Fig. 4b). Similar results, with a trend toward elevated total C4 and significantly increased processed C4, was also seen in CSF from an independent patient cohort (Extended Data Fig. 7). By comparison, complement Factor B, a component of the alternative complement pathway, was not robustly changed in AD CSF (Fig. 4b and Extended Data Fig. 7). Levels of activated subunit Bb were very low in control and AD CSF but did show trends toward increases in AD CSF (Fig. 4b and Extended Data Fig. 7). Thus, the upregulation of glial proteins in the P301S synapse proteome is also present in AD and might be relevant to disease pathophysiology^3,4^. The elevated levels of C4 (and C3 (ref. ^4^)) in CSF from patients with AD are consistent with a role for the CCP in Alzheimer’s neurodegeneration.

### Glial C1q-dependent synapse elimination in P301S mice

**Fig. 5:**
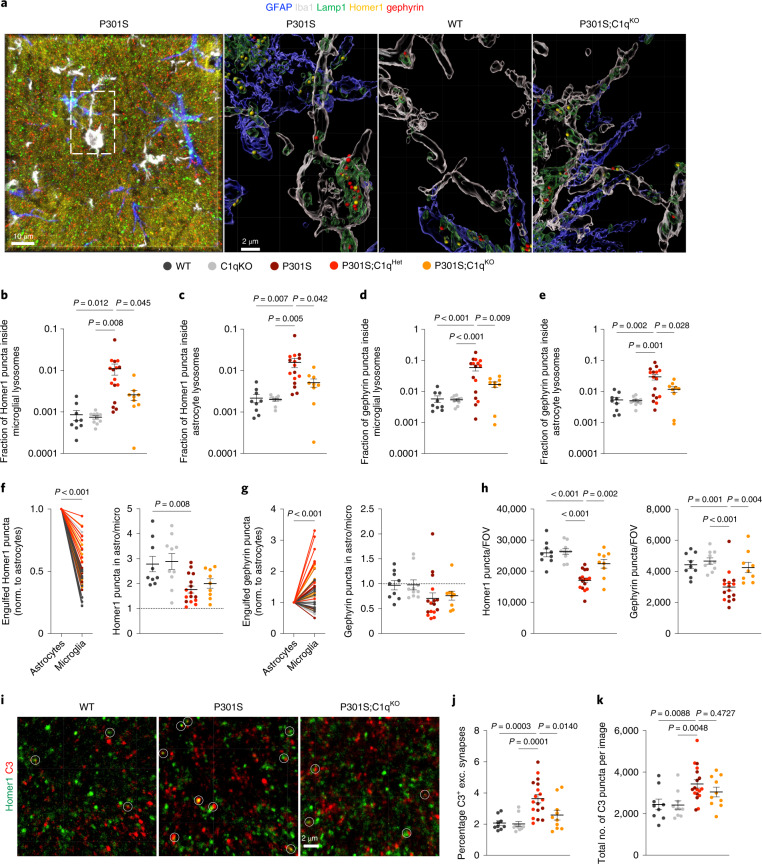
Astrocytes and microglia eliminate excitatory and inhibitory synapses in P301S mice in a complement-dependent manner. **a**, Representative images of a confocal z-stack and Imaris 3D reconstructions of mouse brain sections immunostained for GFAP (blue), Iba1 (white), Lamp1 (green), Homer1 (yellow) and gephyrin (red). LAMP1^+^ lysosomes within GFAP^+^ or Iba1^+^ volumes were classified as astrocytic or microglial lysosomes, respectively. Scale bar in the raw image, 10 µm; scale bar in the Imaris 3D-rendered image, 2 µm. **b**,**c**, Fraction of Homer1 puncta identified inside astrocytic or microglial lysosomes across genotypes. **d**,**e**, Fraction of total gephyrin puncta identified inside astrocytic or microglial lysosomes across genotypes. **f**, Normalized number of Homer1 puncta engulfed by astrocytes or microglia, respectively (left). Ratio of Homer1 puncta within astrocytic/microglial lysosomes (right). **g**, Normalized number of gephyrin puncta engulfed by astrocytes or microglia, respectively (left) and ratio of gephyrin puncta within astrocytic/microglial lysosomes (right). Dotted line in **f** and **g** at a ratio of 1 indicates that astrocytic and microglial lysosomes contained the same number of synaptic puncta, ratio of >1 means that more synaptic puncta were localized within astrocytic lysosomes and <1 indicates that microglial lysosomes contained more synaptic puncta. Connected dots in the left of **f** and **g** show astrocytic and microglial Homer1 or gephyrin engulfment from the same mouse. **h**, Excitatory and inhibitory synapse density across genotypes as measured by number of identified Homer1 and gephyrin puncta per field of view (FOV). **i**, Representative confocal images of immunostained Homer1 (green) and C3 (red) in the CA1 region of WT, P301S and P301S;C1q^KO^ brains. Colocalized Homer1 and C3 puncta are indicated by circles. Scale bar, 2 µm. **j**, Graph shows percentage of C3-labeled Homer1^+^ synapses. **k**, Total number of C3 puncta per FOV. Data were analyzed by one-way ANOVA with Dunnett’s post hoc test (**b**–**e**,**h**,**j**,**k**) and a two-tailed paired Student’s *t*-test (**f**,**g**). Each dot shows average data from one mouse; 7–10 mice per genotype were analyzed. All data are presented as mean ± s.e.m. Source data

**Extended Data Fig. 8:**
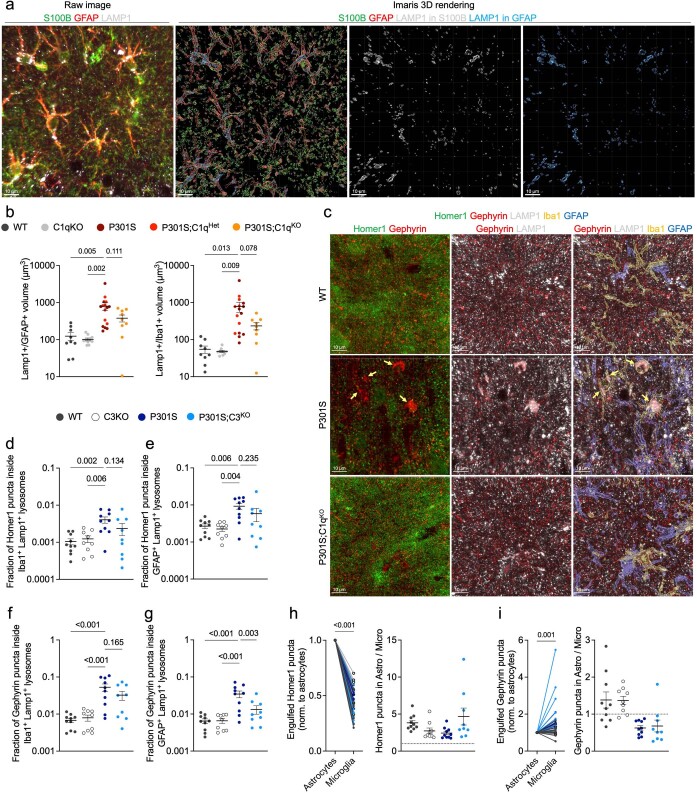
Complement-dependent engulfment of excitatory and inhibitory synapses by astrocytes and microglia in P301S mice. (**a**) Representative image and Imaris 3D rendering of immunostained S100B (green), GFAP (red) and Lamp1 (white). 3D reconstructions show lysosomes (Lamp1 structures) within S100B^+^ astrocytes (white) or GFAP^+^ astrocytes (blue). Note that lysosome structures segmented within S100B^+^ and GFAP^+^ astrocytes are almost identical. Scale bar = 10µm. (**b**) Volume of LAMP1+ lysosomes inside astrocytes and microglia, respectively, in hippocampi from WT, C1q^KO^, P301S and P301S;C1q^KO^. (**c**) Representative images of WT, P301S and P301S;C1q^KO^ brains immunostained for Homer1 (green), Gephyrin (red), LAMP1 (white), Iba1 (yellow) and GFAP (blue). Images on the right show 3D reconstructed GFAP+ astrocytes and Iba1+ microglia together with the raw immunofluorescence from Homer1, Gephyrin and LAMP1. Arrows highlight Gephyrin immunoreactivity accumulated in microglial lysosomes. Note that the neighboring astrocytic lysosomes do contain accumulated Gephyrin. (**d,e**) Fraction of Homer1 and Gephyrin puncta inside astrocytic or microglial lysosomes across genotypes. (**f,g**) Fraction of Gephyrin puncta inside astrocytic or microglial lysosomes across genotypes. (**h**) Normalized number of Homer1 puncta inside astrocytes or microglia, respectively (left graph) and ratio of Homer1 puncta within astrocytic/microglial lysosomes. (**i**) as in H) but showing engulfment data for Gephyrin. Dotted line at a ratio of 1 indicates that astrocytic and microglial lysosomes contained the same number of synaptic puncta, ratio of >1 means that more synaptic puncta were localized within astrocytic lysosomes and <1 indicates that microglial lysosomes contained more synaptic puncta. One-way ANOVA with Dunnett’s post hoc test (B, D-G) or paired two-tailed t-test (H, I). Each dot shows average data from one mouse. In b) 7–10 mice/genotype and in d-i) 9–10 mice/genotype were used. All data are presented as mean ± SEM. Source data

Because of our proteomics, IEM and IHC data, we hypothesized that astrocytes might be interacting with synapses in a C1q-dependent fashion during the process of synapse engulfment. To analyze synapse engulfment by astrocytes and microglia, we immunostained GFAP^+^ astrocytes, Iba1^+^ microglia, Lamp1^+^ lysosomes along with the excitatory postsynapse marker Homer1 (Fig. 5a). As inhibitory synapses are also affected in AD^40^, we additionally immunolabeled the inhibitory postsynaptic marker gephyrin. By confocal microscopy and 3D reconstruction of the hippocampal CA1 area, we measured the amount of Homer1 and gephyrin puncta inside microglial and astrocytic lysosomes within the same image (Fig. 5a). Of note, using GFAP or S100B we identified essentially the same population of astrocytic Lamp1^+^ structures (Extended Data Fig. 8a). As expected from previous studies^3,4^, microglial lysosomes in P301S hippocampi contained excitatory synapses and showed a ~tenfold increase in Homer1 puncta compared to WT controls (Fig. 5b). Compared to P301S, microglial phagocytosis of Homer1 was significantly decreased in P301S;C1q^KO^ brains (Fig. 5b). Notably, we also found a considerable fraction of Homer1 puncta inside astrocytic lysosomes, which was increased ~five- to tenfold in P301S hippocampi (Fig. 5c). The fraction of Homer1 puncta inside astrocyte lysosomes was significantly reduced in P310S;C1q^KO^ brains, indicating that astrocytic eating of excitatory structures in P301S mice was at least in part C1q-dependent (Fig. 5c). Gephyrin puncta were also present in microglial and astrocytic lysosomes (Fig. 5d,e). As with Homer1, eating of gephyrin by microglia and astrocytes was elevated in P301S hippocampi and was partly C1q-dependent (Fig. 5d,e). In healthy brains, however, engulfment of excitatory and inhibitory synapses was unaffected by loss of C1q, as WT and C1q^KO^ hippocampi had the same low amount of Homer1 and gephyrin in glial lysosomes (Fig. 5b–e). The amount of phagocytosed synapse puncta corresponded with changes in the volume of astrocytic and microglial lysosomes across genotypes (Extended Data Fig. 8b).

Notably, we consistently found more Homer1 puncta inside astrocytic versus microglial lysosomes in all genotypes (Fig. 5f). Conversely, gephyrin puncta were less abundant in astrocytic versus microglial lysosomes in P301S hippocampi (regardless of C1q genotype) and similar in astrocytes and microglia in non-transgenic WT animals (Fig. 5g). This microglial proclivity for engulfing gephyrin puncta was particularly notable in P301S brains, where strong accumulation of gephyrin immunoreactivity was often observed in microglial but not astrocytic lysosomes (Extended Data Fig. 8c). One possibility is that this strong immunoreactivity could reflect removal of dendritic segments containing many inhibitory synapses. In line with reduced engulfment of synapses in P301S;C1q^KO^ brains, excitatory and inhibitory synapse loss was ameliorated in C1q-deficient P301S mice (Fig. 5h).

Finally, we tested whether astrocytic eating of synaptic structures requires C3, a central complement component downstream of C1q. The significant increase of Homer1 and gephyrin engulfment by microglia and astrocytes in P301S mice was partially reduced on average in P301S;C3^KO^ mice (Extended Data Fig. 8d–g). However, only the reduction of gephyrin puncta in astrocytic lysosomes reached statistical significance in P301S;C3^KO^ versus P301S mice (Extended Data Fig. 8g), possibly due to high inter-animal variability. Like in the C1q experimental cohort, we found substantially more Homer1 puncta inside astrocytic versus microglial lysosomes in every brain that we analyzed, whereas gephyrin puncta were preferentially found in microglial lysosomes in P301S mice in this C3 experimental cohort (Extended Data Fig. 8h,i).

Next we analyzed C3-labeling of excitatory synapses in the P301S;C1q^KO^ cohort. The percentage of C3^+^ Homer1 puncta was significantly increased in P301S versus WT hippocampi, whereas P301S;C1q^KO^ was comparable to WT hippocampi and significantly decreased compared to P301S brains (Fig. 5i,j). There was no significant difference in the total number of C3 puncta in P301S versus P301S;C1q^KO^ brains (Fig. 5k), indicating that C1q deletion specifically affects C3 deposition at synapses. Overall, the data are consistent with C3 acting downstream of C1q activation and the CCP facilitating synapse elimination by astrocytes and microglia.

### Astrocytes compensate for impaired microglial phagocytosis

Our findings led us to ask, what happens when phagocytic activity is impaired in one of the cell types? Loss-of-function mutations in the microglia-specific *TREM2* strongly increase AD risk^41,42^ and loss of Trem2 in AD mouse models has a profound effect on microglial function and impairs their activation, migration to Aβ plaques and phagocytic activity^43–47^.

**Fig. 6:**
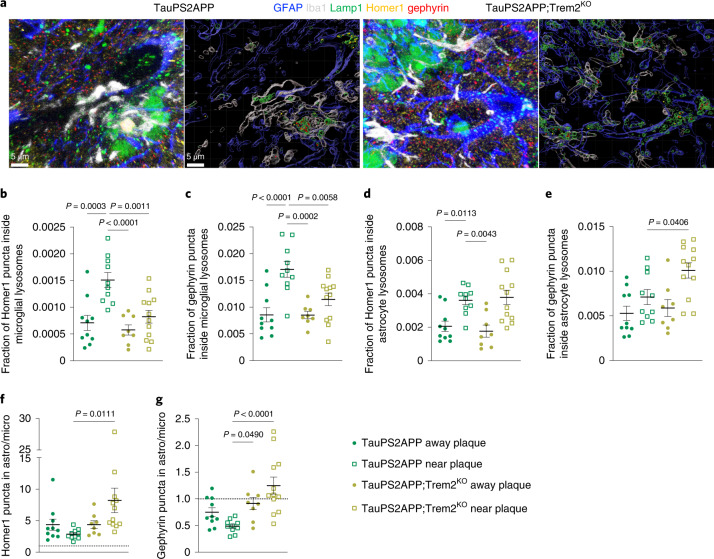
Astrocytes compensate for impaired microglial phagocytosis of inhibitory synapses in Trem2-deficient TauPS2APP mice. **a**, Representative images of confocal z-stack and Imaris 3D reconstructions of mouse brain sections immunostained for GFAP (blue), Iba1 (white), Lamp1 (green), Homer1 (yellow) and gephyrin (red). LAMP1^+^ lysosomes within GFAP^+^ or Iba1^+^ volumes were classified as astrocytic or microglial lysosomes. Plaques were identified indirectly by the presence of large clusters of Lamp1 accumulation (outside of glial cell bodies), which labels dystrophic axons. In the 3D reconstructions (right hand image of each pair), only Lamp1 structures within GFAP or Iba1 volume are rendered. Scale bars, 5 µm. **b**,**c**, Fraction of Homer1 (**b**) or gephyrin (**c**) puncta identified within microglial lysosomes. **d**,**e**, Fraction of Homer1 (**d**) or gephyrin (**e**) puncta identified within astrocytic lysosomes. **f**,**g**, Ratio of Homer1 (**f**) and gephyrin (**g**) puncta within astrocytic/microglial lysosomes. Dotted line at a ratio of 1 indicates that astrocytic and microglial lysosomes contained the same number of synaptic puncta, ratio of >1 means that more synaptic puncta were localized within astrocytic lysosomes and <1 indicates that microglial lysosomes contained more synaptic puncta. Images containing dystrophic axons (plaques), were considered as ‘near plaque’ and images without any dystrophic axons were defined as ‘away plaque’. Data were analyzed by one-way ANOVA with Dunnett’s multiple comparisons test. Each dot shows average data from one mouse; *n* = 10–12 mice per genotype. Note that due to increased plaque load, in some TauPS2APP;Trem2^KO^ mice we were not able to image plaque-free areas. All data are presented as mean ± s.e.m. Source data

To examine whether dysfunctional microglia might result in altered synapse handling by astrocytes, we analyzed the effects of Trem2 deletion in an AD mouse model that combines β-amyloid and Tau pathologies (TauPS2APP)^46^. At 17 months of age, when plaques, phospho-Tau, dystrophic axons and gliosis are present^46^, we immunostained TauPS2APP and TauPS2APP;Trem2^KO^ brain sections using the previously established protocol and imaged hippocampal CA1 regions with and without amyloid plaques (identified by the presence of Lamp1^+^ dystrophic axons) (Fig. 6a). In TauPS2APP brains, microglia and astrocytes phagocytosed more Homer1 and gephyrin puncta near plaques compared to plaque-free areas (Fig. 6b–e). In TauPS2APP;Trem2^KO^ brains, plaque-proximal microglial synapse eating was significantly reduced compared to TauPS2APP mice (Fig. 6b,c), whereas astrocytic eating of Homer1 puncta was unaffected by the lack of Trem2 (Fig. 6d). Notably, astrocytic phagocytosis of gephyrin puncta in the vicinity of plaques was significantly increased in TauPS2APP;Trem2^KO^ versus TauPS2APP brains (Fig. 6e). As in P301S mice, astrocytic lysosomes contained more Homer1 puncta compared to microglial lysosomes (Fig. 6f), whereas gephyrin puncta were more abundant in microglial lysosomes in TauPS2APP mice (Fig. 6g). The overall effect of Trem2-deficiency in TauPS2APP mice was the increase in ratios of both Homer1 and gephyrin inside astrocytic versus microglial lysosomes (Fig. 6f,g). Thus, Trem2 is necessary for efficient synapse engulfment by microglia near plaques and astrocytes can, at least in part, compensate for impaired microglial phagocytosis of inhibitory synapses.

## Discussion

Multiple lines of evidence support the idea that overactivation of the CCP contributes to synapse loss and neuronal damage in AD^3,4,15,17^. Here, we found that C1q deletion was protective against neurodegeneration without preventing gliosis, Tau pathology or gross transcriptomic changes, implying C1q and CCP act downstream of Tau pathology and gliosis. We provide a deep proteomic dataset that catalogs synaptic protein changes as well as altered glial protein association with synapses. Our data show a new role for astrocytes in complement-dependent removal of excitatory and inhibitory synapses. These follow-up experiments also indicated concomitant/coordinated roles for astrocytes and microglia in synapse engulfment during pathophysiology.

Although functional benefits upon C1q deletion in P301S mice (and C3 deletion in P301S and PS2APP mice^4^) suggest complement-dependent glial elimination of functional synapses, one limitation of our study is that by immunostaining and analysis of fixed brains, we could not distinguish between glial pruning mechanisms and cleaning of debris. That technical caveat noted, we found that while astrocytic lysosomes contained more Homer1 puncta, gephyrin immunoreactivity was preferentially found in microglial lysosomes, revealing an unexpected ‘division of labor’ between astrocytes and microglia^20^. In TauPS2APP;Trem2^KO^ mice where microglial synaptic engulfment was impaired, astrocyte phagocytosis partially compensated for the engulfment of gephyrin puncta, possibly because inhibitory synapses are normally predominantly engulfed by microglia. Together with a recent study that identified astrocytic removal of synapses in the adult brain using fluorescent phagocytosis reporters^20^, our study identifies that astrocytes are a key phagocyte of synapses.

In contrast to the C1q-dependent synapse engulfment in P301S mice, we find that the basal levels of astrocytic (and microglial) synapse engulfment in healthy brains is C1q-independent. One explanation for this difference could be the low expression of complement genes^4^ (and low basal complement activity) in the healthy adult brain. Alternatively, disease-associated astrocytes might induce the expression of (yet unknown) complement receptor(s). Microglial removal of synapses is mediated by microglial CR3 recognition of C3b deposited on synapses^15,18^; however, astrocytes do not express CR3 as microglia do. Phosphatidylserine (PS) has been identified as an ‘eat-me’ signal that labels synapses for microglial removal during development as well as plaques in AD models^48,49^. Potentially, the increase in annexin family proteins in human AD and P301S synapse fractions might reflect their binding to damaged, PS-exposing synaptic membranes, whereas their reduction in P301S;C1q^KO^ synapses may reflect less damaged synapses in C1q-deficient brains. MERTK and Megf10 are astrocyte-expressed phagocytosis receptors that bind exposed PS via its ligands Gas6 and Protein S and mediate engulfment of synapses under physiological conditions^20,21^. Megf10 has been shown to bind C1q and thereby mediate clearance of apoptotic cells by astrocytes^50^ and hence might be involved in astrocytic synapse eating; however, none of the known glial phagocytic receptors was identified in our synapse fractions. Future experiments will need to determine whether specific astrocytic receptors directly detect complement deposition on neurons or whether there may be indirect complement-dependent signaling that triggers local activation of astrocyte processes around synapses.

Microglia have previously been shown to prune inhibitory synapses in physiological and pathological conditions in a PS- and complement-dependent manner, respectively^11,51^. GABA_B_ receptors expressed in a subset of microglia facilitate microglial pruning of inhibitory synapses during circuit development^52^ and GABA-receptive microglia might also engulf inhibitory synapses in a disease context. Given that astrocytes and microglia eliminate synapses in a C1q/complement-dependent manner in P301S mice it is possible that they ‘compete’ for the same synapses that are destined for removal or phagocytosis of synapses might be orchestrated between astrocytes and microglia, like the removal of apoptotic cells^53^. In ischemia, phagocytic activity of astrocytes and microglia show spatiotemporal differences^54^, suggesting that there might be astrocyte–microglia coordination in remodeling of damaged tissue. In this context, it is notable that in MS mouse models, microglia but not astrocytes eliminate synapses through the alternative complement pathway^12^. Thus, it is possible that astrocytes and microglia sense different complement molecules at the synapse.

Overall, our results greatly advance our understanding of the mechanisms underlying complement-mediated synapse elimination and neuronal damage, identify an unexpected division of labor of inhibitory versus excitatory synapse phagocytosis between microglia and astrocytes and open new avenues into potential therapeutic approaches for AD.

## Methods

### Mice

P301S mice (expressing human Tau with the P301S mutation, driven by the PrP promoter^55^), were crossed to C1qC KO mice (Jax, 029409). All P301S mice were hemizygous for the TauP301S transgene and cohorts were produced with all genotypes as littermates. The TauPS2APP model was generated as previously described by crossing PS2APP mice with mice expressing the P301L mutant human Tau protein and all experimental animals were homozygous for PS2APP and hemizygous for the P301L transgene^46^. As previously described, TauPS2APP mice were crossed with mice carrying the Trem2tm1(KOMP)Vlcg null allele^46^. Throughout the study we analyzed male mice. All animal studies were authorized and approved by the Genentech Institutional Animal Care and Use Committee. Mice were group-housed up to five mice per cage in individually ventilated cages within animal rooms maintained on a 14:10-h, light–dark cycle. Animal rooms were temperature and humidity-controlled, between 20–26 °C and 30–70%, respectively, with 10–15 room air exchanges per hour. Mice had ad libitum access to water and food. All testing occurred during the light phase.

### Human CSF samples

CSF from patients with AD and healthy controls was obtained from Folio Biosciences and Precision Medicine with ethics committee approval and written informed consent. The patient cohorts were described previously^4^.

### Volumetric brain MRI

MRI was performed on a 9.4T Bruker system with a four-channel receive-only cryogen-cooled surface coil and a volume transmit coil (Bruker). T2-weighted images were acquired with a multi-spin echo sequence: TR 5,100 ms; TE 10, 20, 30, 40, 50, 60, 70 and 80 ms; 56 contiguous axial slices of 0.3 mm thickness; FOV 19.2 mm × 19.2 mm; matrix size 256 × 128, 1 average, with a scan time of 11 min per mouse. During imaging, anesthesia was maintained at 1.5% isoflurane and body temperature was maintained at 37 ± 1 °C using a feedback system with warm air (SA Instruments). The regional and voxel differences in the brain structure were evaluated by registration-based region of interest analysis. In brief, multiple echo images were averaged and corrected for field inhomogeneity to maximize the contrast-to-noise ratio and images were analyzed based on a 20-region predefined in vivo mouse atlas (https://github.com/dmac-lab/mouse-brain-atlas) that was co-registered to a study template and warped to individual mouse datasets. All the co-registration steps were performed in SPM8 (Wellcome Trust Centre for Neuroimaging, UCL).

### Behavior/open field

Spontaneous locomotor activity was measured with an automated Photobeam Activity System-Open Field (San Diego Instruments). Mice were placed individually in a clear plastic chamber (41 cm length × 41 cm width × 38 cm height) and their horizontal and vertical movements were monitored for 15 min per session with two 16 × 16 photobeam arrays.

### Histology and analysis

Mice were deeply anesthetized and transcardially perfused with phosphate-buffered saline (PBS). Hemi-brains were drop-fixed for 48 h at 4 °C in 4% paraformaldehyde. After being cryoprotected and frozen, up to 40 hemi-brains were embedded per block in a solid matrix and sectioned coronally at 30 μm (MultiBrain processing by NeuroScience Associates) before being mounted onto slides.

#### Immunohistochemistry

Brain sections were stained for AT8 (Thermo Scientific MN1020B, 1:5,000 dilution), GFAP (Dako Z0334, 1:20,000 dilution), Iba1 (Abcam ab178846, 1:100,000 dilution), NeuN (Millipore MAB377B, 1:1,500 dilution) and Amino Cupric (using established protocols as described previously^4^). Brightfield slides processed by NeuroScience Associates were imaged on the Leica SCN400 whole-slide acquisition system (Leica Microsystems) at ×200 magnification. Quantification of chromogenic staining area was performed using grayscale and color thresholds followed by morphological operations. Positive stain area was normalized to the whole brain section or the manually marked up hippocampal area.

#### Immunofluorescence measurement of C1q levels

Free-floating sections in PBS with 0.1% Triton X-100 (PBST), were blocked with 5% normal donkey serum in PBST (NDST) and incubated overnight at 4 °C with primary antibody in 1% NDST. Secondary antibodies in 1% NDST were incubated for 2–3 h at room temperature, washed in PBST and PBS and mounted using NeuroScience Associates Mounting Solution pH 6.0 (NeuroScience Associates). Slides were cover-slipped with ProLong Diamond Anti-fade Mountant with DAPI. Primary antibody was C1q (1:1,000 dilution clone 4.8, rabbit monoclonal, Abcam ab182451). Alexa Fluor secondary antibody goat anti-rabbit IgG (H+L) Highly Cross-Adsorbed Secondary Antibody, Alexa Fluor 594 (Thermo Fisher, A11012) was used at 1:500 dilution. Immunofluorescent slides were imaged at ×200 magnification using the Nanozoomer-XR (Hamamatsu) whole-slide scanner equipped with a fluorescent imaging module and standard filter wheel. All whole-slide image analysis was performed in a blinded manner using MATLAB v.9.4 (Mathworks). Total tissue area was detected by thresholding on the DAPI and Alexa-594 signal and merging and processing of the binary masks by morphological operations. Hippocampal regions of interest were marked up manually. Pixel intensity was evaluated in 8-bit grayscale and the C1q integrated pixel intensity in the whole-brain section or hippocampus was normalized to the whole tissue or hippocampal area, respectively. Data were averaged from two sections per animal.

### CSF total and processed complement assays

C4 and processed C4, Factor B and processed Factor B were measured in human CSF using custom single molecule array (Simoa) assays (Quanterix). For the C4 assay, the main reagents consisted of paramagnetic carboxylated beads (Quanterix) coated with a rabbit anti-C4 antibody (abx102219, Abbexa) and a biotinylated mouse anti-C4c detection antibody (A211, Quidel). For processed C4, which measures the C4c protein fragment, the main reagents consisted of paramagnetic carboxylated beads (Quanterix) coated with a mouse anti-C4c antibody (C7850-18B1, US Biological) and a biotinylated mouse anti-human C4 (LS-C128299, LSBio). Conjugations were performed using the standard recommended concentrations and challenge ratios from Quanterix. For the Factor B (FB) assay, the main reagents consisted of paramagnetic carboxylated beads (Quanterix) coated with a mouse anti-FB antibody (ab17927, Abcam) and a biotinylated anti-Bb detection antibody (Genentech). For processed Factor B, which measures the Bb protein fragment, the main reagents consisted of paramagnetic carboxylated beads (Quanterix) coated with an anti-Bb antibody (Genentech; same antibody for FB capture) and a biotinylated mouse anti-human Bb (A252, Quidel). Conjugations were performed using the standard recommended concentrations and challenge ratios from Quanterix.

Assays were run using one of the standard protocols for the Simoa HD-1 instrument from Quanterix^4^. In the protocol, 25 μl of capture-coated beads were incubated for 30 min with 25 μl of diluted sample. After washing, immunocomplexes were incubated for 5 min with 100 μl of the biotinylated detection antibody. Washed immunocomplexes were incubated for 5 min with 100 μl of streptavidin-conjugated β-galactosidase (Quanterix). After a last round of washes, the beads were resuspended in resorufin β-d-galactopyranoside (Quanterix) and the mixture was then applied to Simoa disks. The HD-1 analyzer was used to read the resulting fluorescent signal and calculate the average number of enzymes per bead (AEB) for tested samples. The reported AEB values were analyzed against a calibrator curve constructed by AEB measurements on native human C4 (A105, Complement Technology, C4 assay) or C4c (32R-AC050, Fitzgerald, PC4 assay) or Factor B (A135, Complement Technology, FB assay) or Bb (A155, Complement Technology, Bb assay) protein serially diluted in assay diluent. Samples were analyzed using a single batch of reagents and testing across three runs. For each of the three runs, the PC4 and C4 or FB and Bb assays were run together with calibrators and controls for each assay and an approximately equal number of samples from healthy individuals and patients with AD samples were tested at the chosen dilutions.

### Bulk RNA-seq

Ten-month-old WT (*n* = 4), C1q^KO^ (*n* = 4), P301S (*n* = 4) or P301S;C1q^KO^ mice were perfused with cold PBS and the hippocampi were immediately sub-dissected and preserved in RNAlater. RNA was extracted from samples using QIAGEN RNeasy Plus Mini kit. The concentration of RNA samples was determined using a NanoDrop 8000 (Thermo Scientific) and RNA integrity was determined by Fragment Analyzer (Advanced Analytical Technologies). Then, 0.5 μg of total RNA was used as an input material for library preparation using TruSeq RNA Sample Preparation kit v2 (Illumina). Library size was confirmed using a Fragment Analyzer (Advanced Analytical Technologies). Library concentrations were determined by qPCR-based using a Library quantification kit (KAPA). The libraries were multiplexed and then sequenced on Illumina HiSeq2500 (Illumina) to generate 30 M of single-end 50-bp reads per library^56^.

The fastq sequence files for all RNA-seq samples were filtered for read quality (keeping reads where at least 70% of the cycles had Phred scores ≥23) and ribosomal RNA contamination. The remaining reads were aligned to the mouse reference genome (GRCm38) using the GSNAP alignment tool^57^. These steps and the downstream processing of the resulting alignments to obtain read counts were implemented in the Bioconductor package HTSeqGenie (https://bioconductor.org/packages/release/bioc/html/HTSeqGenie.html). Only uniquely mapped reads were used for further analysis. Differential gene expression analysis was performed with voom + limma^58^ (only C1qc was significant in the KO versus WT comparison, so further results are not shown in the text).

For heat maps (Extended Data Figs. 2 and 5c), gene expression data were first normalized to nRPKM statistic as described^58^, then transformed to a log_2_ scale. Any values less than −40 were then replaced by −40 and a standard *z* score calculation was performed (for each gene, subtracting mean and dividing by s.d.) and then used for visualization.

### Single-cell RNA-seq

Nine-month-old WT (*n* = 3) or P301S^het^ (*n* = 6) mice were perfused with cold PBS and the hippocampi were immediately sub-dissected. Single-cell suspensions were prepared from the hippocampi as described elsewhere^46^. Briefly, hippocampi were chopped into small pieces and dissociated with enzyme mixes in a Neural Tissue Dissociation kit (P) (Miltenyi, 130-092-628) in the presence of actinomycin D. After dissociation, cells were resuspended in Hibernate A Low Fluorescence medium (Brainbits) containing 5% FBS, with Calcein Violet AM (Thermo Fisher, C34858) and propidium iodide (Thermo Fisher, P1304MP). Flow cytometry was used to sort and collect live single-cell suspensions for the scRNA-seq study.

Sample processing and library preparation was carried out using the Chromium Single Cell 3′ Library and Gel Bead kit v3 (10x Genomics) according to the manufacturer’s instructions. Cell-RT mix was prepared to aim for 10,000 cells per sample and applied to Chromium Controller for gel bead-in-emulsion generation and barcoding. Libraries were sequenced with HiSeq 4000 (Illumina). scRNA-seq data were processed with an in-house analysis pipeline as described previously^46,59^. Reads were demultiplexed based on perfect matches to expected cell barcodes. Transcript reads were aligned to the mouse reference genome (GRCm38) using GSNAP (2013-10-10)^57^. Only uniquely mapped reads were considered for downstream analysis. Transcript counts for a given gene were based on the number of unique molecular identifiers (UMIs) (up to one mismatch) for reads overlapping exons in sense orientation. Cell barcodes from empty droplets were filtered by requiring a minimum number of detected transcripts. Sample quality was further assessed based on the distribution of per-cell statistics, such as total number of reads, percentage of reads mapping uniquely to the reference genome, percentage of mapped reads overlapping exons, number of detected transcripts (UMIs), number of detected genes and percentage of mitochondrial transcripts. After this primary analysis step, cells with less than 1,000 total UMIs or greater than 10% mitochondrial UMIs were discarded. UMI normalization was performed by dividing each gene expression value for a cell by a factor proportional to the total number of transcripts in that cell. Letting *n*_*c*_ represent the total number of UMIs for cell *c*, then the normalization factor *f*_*c*_ for that cell was given by

$$f_c = \frac{{n_c}}{{median_{c\prime }(n_{c\prime })}}$$

(with c′ going over all cells) and the ‘normalized UMIs’ for gene *g* and cell *c* given by *nUMI*_*g,c*_ = *n*_*c*_ / *f*_*c*_^60^.

Pseudobulk microglial, astrocyte, oligodendrocyte and neuron expression profiles were derived from single-cell datasets first by aggregating each sample’s data for each cell type as described^46^. A single ‘raw count’ expression profile was created for each pseudobulk simply by adding the total number of UMIs for each gene across all each cell of that type from that sample. This gave a gene-by-pseudobulk count matrix, which was then normalized to a normalizedCount statistic using the estimateSizeFactors function from DESeq2 (ref. ^61^), used for calculating gene set scores and visualizing gene expression and for normalization factors for DE analysis. DE was performed on pseudobulk datasets using voom + limma methods for bulk RNA-seq. To put this into more formal notation, let n_*ij*_ be the raw UMI number of gene *i* in each cell type *j*. Let s_*j*_ indicate the sample of cell *j*. The pseudobulk count matrix B, with rows indexed by genes and columns indexed by samples (instead of cells) is defined as

$$B_{is} = \mathop {\sum}\limits_{j:s_j = s} {n_{ij}}$$

The matrix is then size-factor normalized and analyzed using the standard methods of bulk RNA-seq, including DE analysis using voom + limma^58^. Finally, the normalized pseudobulk expression matrix was then used to construct Fig. 3A and Extended Data Fig. 5a,f,h by calculating the average percentage of gene expression in each cell type (excitatory neurons, astrocytes, microglia and oligodendrocytes) in P301S mice.

### PSD/synapse fraction isolation and mass spectrometry analysis

Synapse fractions were isolated as previously described with minor modifications^3^. Briefly, dissected hippocampi in ice were homogenized in cold buffer (5 mM HEPES (pH 7.4), 1 mM MgCl_2_, 0.5 mM CaCl_2_ supplemented with phosphatase and protease inhibitors) with a Teflon homogenizer. After 1,400*g*, 10 min centrifugation at 4 °C the supernatant was pelleted by centrifugation (13,800*g*, 10 min at 4 °C). The pellet was resuspended in 0.32 M Tris-buffered sucrose and ultra-centrifuged into a 1.2, 1 and 0.85 M sucrose gradient at 82,500*g* for 2 h at 4 °C. The synaptosome fraction between the 1 M and 1.2 M sucrose interface was carefully collected, the same volume of 1% Triton X-100 was added, then mixed and incubated on ice for 15 min. The final synapse fraction was pelleted at 32,800*g* for 20 min at 4 °C. For each age, a cohort of 11 samples was analyzed. For the 6-month-old group of mice, we isolated synapse fractions from three WT, three P301S, two C1q^KO^ and three P301S;C1q^KO^ hippocampi. Due to hippocampal atrophy and to reduce inter-animal variability, we pooled hippocampi in the 9-month-old cohort. Each pool contained samples from two mice (WT, C1qKO and P301S;C1q^KO^) or four mice (P301S), respectively.

Enriched PSD samples were adjusted to a pH of 8.5 before reduction (5 mM dithiothreitol, 45 min at 37 °C), alkylation (15 mM IAA, 30 min at room temperature in the dark) and capping (5 mM dithiothreitol, 15 min at room temperature in the dark). Proteins were digested by LysC (1:50 ratio of enzyme to substrate) for 3 h at 37 °C before digestion with trypsin (1:50 ratio of enzyme to substrate) O/N at room temperature while shaking. Peptides were acidified, desalted using the Phoenix peptide cleanup kit (PreOmics) and dried before quantification with a peptide BCA kit (Thermo Fisher). Peptides were labeled with TMT multiplexing reagents (Thermo Fisher) according to the manufacturer’s instructions. Following labeling with isobaric tags, the samples were mixed, dried and desalted before fractionation. For young mouse samples, peptides were separated by offline high-pH reversed-phase fractionation using an ammonium formate-based buffer system delivered by an 1100 HPLC system (Agilent). Peptides were separated over a 2.1 × 150 mm, 3.5 µm 300Extend-C18 Zorbax column (Agilent) and separated over a 75-min gradient from 5% ACN to 85% ACN into 96 fractions. The fractions were then pooled into 24 tubes, of which 12 were analyzed. For old mouse samples, peptides were fractionated using a high-pH spin cartridge (Pierce Thermo Fisher) where 16 fractions were collected and concatenated into eight final fractions, all of which were analyzed. Following fractionation, peptides were dried and desalted a final time by stage tip.

Samples were analyzed on an Orbitrap Fusion Lumos mass spectrometer (Thermo Fisher) coupled to an Ultimate 3000 RSLCnano ProFlow HPLC system (Thermo Fisher). Peptides were separated over a 100 µm × 250 mm PicoFrit column (New Objective) packed with 1.7 µm BEH-130 C18 (Waters) at a flow rate of 450 nl min^−1^ or over a 25-cm IonOpticks Aurora column (IonOpticks) at 300 nl min^−1^ for a total run time of 180 min. The gradient started at 2 or 5% B (98% ACN and 1% FA) and ended at 30% B over 140 min and then to 50% B at 160 min. Orbitrap MS1 survey scans (120,000 resolution, AGC = 1 × 106 and maxIT = 50 ms) were used to select the top ten most intense precursors, ensuring that only one charge state per precursor (±10 ppm) was selected once every 45 s. Selected peptides were fragmented by CAD (normalized collision energy = 35) and analyzed in the ion trap (AGC = 2 × 104, maxIT = 100 ms) for identification. For quantification, the eight most intense peaks from the MS2 were selected for SPS-MS3 analysis where peptide fragments were re-isolated and fragmented (higher-energy collisional dissociation and normalized collision energy = 55) and analyzed in the Orbitrap (50,000 resolution, AGC = 2.2 × 105 or 2.5 × 105, maxIT = 150 ms or 200 ms).

Mass spectral data were assigned to peptides using a concatenated target-decoy database consisting of mouse sequences and common laboratory contaminants from UniProt (v.2016-06) using Mascot (Matrix Science) with a 25-ppm precursor ion mass tolerance, 0.8-Da fragment ion tolerance, a fixed proprionamide modification on all cysteines, a fixed modification of the TMT six-plex reagent on lysine and peptide amino termini, a variable modification of the TMT six-plex reagent on tyrosine and a variable oxidation modification on methionine, full tryptic specificity and a maximum of one missed cleavage. Peptide-spectral matches were filtered to a false discovery rate (FDR) of 5% at the peptide level using a linear discriminant approach and subsequently filtered to a 2% protein FDR.

Quantification and statistical testing of TMT proteomics data was performed using MSstats v.3.14.1 (ref. ^62^). Before MSstats analysis, peptide-spectral matches (PSMs) were filtered to remove matches from decoy proteins; peptides with length less than 7; isolation specificity <50%; reporter ion intensity <256; and summed reporter ion intensity (across all channels) <30,000. In the case of redundant PSMs (multiple PSMs in one MS run that map to the same peptide), PSMs were summarized by the maximum reporter ion intensity per peptide and channel and median equalized. In the case of redundant PSMs across fractions (redundant matching PSMs being found in multiple fractionated runs), PSMs were summarized by selecting the fraction with the maximum reporter ion intensity for each PSM. Protein level summarization was performed using a Tukey median polish approach. Differential abundance analyses between conditions were performed in MSstats based on a linear mixed-effects model per protein.

### Pathway analysis and protein subcellular location

Pathway enrichment analysis was performed using ShinyGO^63^. Only up- and downregulated DE proteins were included in the analysis. Enriched KEGG pathways with an FDR < 0.05 were considered statistically significant and selected KEGG pathways are represented with their respective FDR values.

UniProt annotation and GO cellular component was used to define protein subcellular location^64^. Primary literature search was used to validate the Uniprot-annotated subcellular location of selected proteins.

### LD-score regression

We applied stratified LD-score regression (S-LDSC)^30^ to evaluate polygenic enrichment in differentially abundant proteins in the P301S, P301S;C1q^KO^ and C1q^KO^ PSDs. First, we mapped proteins from the mouse proteome to their corresponding genes using Uniprot Knowledgebase (https://www.uniprot.org/id-mapping) and converted the mouse genes to their human orthologs using the NCBI HomoloGene database. S-LDSC had primarily been used to analyze enrichment of large gene sets and it was shown that a Type I error is not always controlled in the analysis of small annotations or gene sets^65^. Therefore, we defined two protein sets of the top 1,000 upregulated and 1,000 downregulated proteins in each PSD proteome. In brief, S-LDSC tested whether the heritability explained by SNPs near a set of genes or a specific genomic annotation was significantly greater than expectation. The model estimated significance after correcting for LD structure and controlling for genomic properties that include epigenetic marks, evolutionary conservation and protein-coding regions (as defined in the S-LDSC baseline model). We focused our analysis on HapMap single-nucleotide polymorphisms (SNPs) that were 100 kb up- and downstream of each protein in the protein set. We tested for enrichment using the GWAS summary statistics from 752 traits (629 from the UK Biobank traits and 123 traits from other genetic studies)^28–34^. The UK Biobank traits selected were demonstrated to have a significant, non-zero heritability as estimated using LD-score regression. We used Bonferroni correction across 752 traits at *α* = 0.05 (multiple corrections adjusted *P* threshold  0.05 / 752 = 0.000066) to define significantly enriched traits. As performed in other analyses of UK Biobank phenotypes, we assigned each trait to one of 24 domains to identify enrichment trends among similar phenotypes^66^.

### Immuno-electron microscopy and quantitative analysis

IEM analysis was performed on hippocampus sections that were prepared previously^3^. Briefly, mice were anesthetized and perfused with PBS followed by 4% PFA fixative. The brains were then cut in 1-mm thick sagittal sections and post-fixed overnight in 4% PFA. The tissue slices were rinsed in PBS and PBS with 0.15% glycin, embedded in flat slabs of 12% gelatin in 0.1 M phosphate buffer (PB) and cryoprotected with 2.3 M sucrose in 0.1 M PB. The hippocampus was excised from the brain slices and cut in an anterior and posterior half, each ~1 mm^3^ in size. Each block was mounted on an aluminum pin such that sagittal hippocampus sections could be cut with known ventral–dorsal orientation and frozen in liquid nitrogen. From these blocks, ultrathin cryosections were cut at −120 °C on cryo-ultramicrotomes Leica EM UC6 and UC7 with attached cryo-chamber FC6 and FC7 (Leica Microsystems), thawed and placed on copper carrier grids. The grids with sections were sequentially incubated in PBS at 37 °C to dissolve gelatin, then at room temperature with guinea pig anti-EAAT2/GLT-1 antibody (Millipore, AB1783, 1:300 dilution), followed by 10 nm Protein A-gold particles (Cell Microscopy Core, University Medical Center Utrecht), both in blocking solution. The blocking solution contained 0.5% fish skin gelatin (Sigma, G7765), 0.1% acetylated BSA (Aurion, 900.099) and 1% BSA (Sigma, A4503) in PBS. Final staining of the sections was performed with uranyl acetate (SPI-Chem, 02624-AB) followed by a uranyl acetate-methylcellulose (Sigma, M-6385) mixture. To retrace the CA1 and DG regions in the sections of the posterior half of the hippocampus in electron microscopy, serially sectioned semi-thin cryosections deposited on glass slides were stained sequentially with toluidine blue (Sigma-Aldrich, T3260, 1% in distilled water) and methylene blue-borax-azur(II) (Merck, 101283, 106308 and 109211, respectively, each 0.5% in distilled water). The light microscopy and electron microscopy images were then correlated.

For the quantitative analysis of synapses and their association with astrocytes, EAAT2/GLT-1-labeled sections were examined in a JEM-1011 transmission electron microscope (JEOL) equipped with a Veleta Megaview G2 CCD camera with Radius software (EMSIS). Images of synapses at ×60,000 magnification were collected in a systematic random way on sections from three WT and three P301S mice, separately in the CA1 and DG regions. From each synapse profile, recognizable by a synaptic cleft, a PSD in the spine and synaptic vesicles in the axon terminal, the perimeter length was measured. In addition, the length of all GLT-1-positive astrocyte plasma membrane segments directly facing the plasma membrane of each synapse was measured. Membrane lengths were quantified using Fiji software. Average values for 10–17 synapses per hippocampal region for each mouse were calculated.

### Astrocyte–Homer1 association, synapse engulfment imaging and analysis

#### Synapse engulfment analysis

Free-floating sections were incubated with PBS with 0.2% Triton X-100 (PBST) and 10% normal goat serum for 1 h at room temperature. After blocking, sections were incubated with primary antibodies in PBST at 4 °C for 16–24 h. The following primary antibodies were used: mouse anti-GFAP (1:1,000 dilution, clone ASTRO6, Thermo Fisher), rabbit anti-Iba1 (1:1,000 dilution, polyclonal, Wako), rat anti-LAMP1 (1:250 dilution, BioLegend), chicken anti-Homer1 (1:1,000 dilution, Synaptic Systems) and guinea pig anti-gephyrin (1:750 dilution, Synaptic Systems). After three washes with PBST, sections were incubated with a secondary antibody cocktail consisting of goat anti-mouse IgG (H+L) Highly Cross-Adsorbed Secondary Antibody, Alexa Fluor Plus 405 (Thermo Fisher, A48225), goat anti-rat IgG H&L (Alexa Fluor 488) preadsorbed (Abcam, ab150165), goat anti-chicken IgY H&L (Alexa Fluor 555) preadsorbed (Abcam, ab150174), goat anti-guinea pig IgG (H+L) Highly Cross-Adsorbed Secondary Antibody, Alexa Fluor 633 (Thermo Fisher, A21105) and goat anti-rabbit IgG (H+L) Highly Cross-Adsorbed Secondary Antibody, Alexa Fluor 680 (Thermo Fisher, A21109), all 1:1,000 dilution in PBST for 1 h at room temperature. After a wash with the second antibody, sections were mounted with anti-fade reagent (ProLong Diamond Invitrogen). Digital images were acquired using a ×100 (NA 1.4) oil-immersion objective on a Leica SP8 laser scanning confocal microscope using 405 nm, 488 nm, 555 nm, 633 nm and 680 nm excitation wavelengths for collecting corresponding Alexa fluorescence signals. Synapse engulfment analysis was performed as previously described^4^. First, Iba1^+^ microglia and GFAP^+^ astrocytes were 3D-reconstructed using the surface-rendering function. Next Lamp1^+^ lysosomes within microglia and astrocytes, respectively, were segmented using the surface-rendering function. Homer1 and gephyrin puncta were identified using the spots function and classified a lysosomal versus non-lysosomal using the minimal distance function. In TauPS2APP mice, plaques were identified indirectly by the presence of Lamp1 accumulation, which labels dystrophic axons. Z-stacks with *xy* dimensions of 93.1 × 93.1 µm containing dystrophic axons (plaques), were considered as ‘near plaque’ and images without any dystrophic axons were defined as ‘away plaque’. Fraction of lysosomal Homer1 and gephyrin puncta were calculated by dividing lysosomal puncta/non-lysosomal puncta. A total of 4–5 images containing multiple microglia and astrocytes in the CA1 region were analyzed per mouse.

#### Astrocyte–Homer1 association

For the astrocyte–Homer1 association, free-floating brain sections were immunostained as described above. The following primary antibodies were used: mouse anti-GFAP (1:1,000 dilution, clone ASTRO6, Thermo Fisher), mouse anti-S100B (1:750 dilution, Abcam) and chicken anti-Homer1 (1:1,000 dilution, Synaptic System), followed by incubation with Alexa-conjugated secondary antibodies. Digital images were acquired using a ×100 oil-immersion objective on a Leica SP8 laser scanning confocal microscope or a ×60 oil-immersion objective on an Andor DragonFly spinning disk confocal microscope. Confocal stacks were analyzed using Imaris 9.6.1. For astrocyte–Homer1 associations, the GFAP or S100B channel was subjected to Gaussian filtering and background subtraction. GFAP^+^ or S100B^+^ astrocytes were 3D-reconstructed using the surface-rendering function. Homer1 puncta were reconstructed using the spots function and their total number was calculated. Next, the number of Homer1 puncta located up to 0.3 µm from the astrocyte surface was identified and considered as Homer1 puncta associated with astrocytes. Percentage of astrocyte-associated Homer1 puncta was calculated by dividing it with the total number of Homer1 puncta in each image.

#### Homer1–C3 colocalization

Free-floating brain sections were immunostained as described above with the following primary antibodies: rabbit anti-C3 (1:750 dilution, Dako/Agilent A063) and chicken anti-Homer1 (1:1,200 dilution, Synaptic System). Images were acquired using a ×60 oil-immersion objective on an Andor DragonFly spinning disk confocal microscope. Colocalization was calculated using the Fiji ComDet plugin (https://github.com/ekatrukha/ComDet). Briefly, images were convoluted with a Gaussian Mexican hat filter using an approximate puncta size of 3 pixels (1 pixel = 100 nm). Puncta were identified using an intensity threshold of 3 × s.d. for Homer1 and 6 × s.d. for C3. Puncta were considered as colocalized if the max distance between the spots’ centers was <3 pixels (300 nm).

### Statistics and reproducibility

Experimenters were blind to genotype for all behavioral measurements, microscopic and histological analyses. No specific methods were used to randomly allocate samples to groups. No statistical method was used to predetermine sample size, but our samples sizes are similar to those reported in previous publications^3,4^. No data were excluded from the analyses. Statistical analyses were performed with GraphPad Prism software v.9 (GraphPad Software). All parameters were expressed as mean ± s.e.m., unless otherwise stated. Data distribution was assumed to be normal but this was not formally tested. Two-by-two group comparisons were analyzed using two-way ANOVA followed by post hoc tests (stated in the figure legends). For comparison of two groups, a two-tailed Student’s *t*-test was used. For multiple groups, one-way ANOVA followed by a post hoc test (listed in the figure legends) was used.

### Reporting summary

Further information on research design is available in the Nature Research Reporting Summary linked to this article.

## Supplementary information

Reporting Summary.

Supplementary Table 1 Quantitative proteomics data of the hippocampal synapse proteomes from 6- and 9-month-old mice.

## Data Availability

Source data are provided with this paper and all other data are available from the authors upon request. The following datasets have been deposited to public repositories: scRNA-seq, including P301S mice (Gene Expression Omnibus accession no. GSE180041); bulk RNA-seq P301S × C1qKO (Gene Expression Omnibus accession no. GSE186414); and proteomics data, MassIVE (10.25345/C58P18). Other datasets used for analysis are publicly available and include the mouse reference genome (GRCm38), GCA_000001635.2.

## Source data

Source Data Fig. 1 Numerical source data.
Source Data Fig. 2 Numerical source data.
Source Data Fig. 3 Numerical source data.
Source Data Fig. 4 Numerical source data.
Source Data Fig. 5 Numerical source data.
Source Data Fig. 6 Numerical source data.
Source Data Extended Data Fig. 1 Numerical source data.
Source Data Extended Data Fig. 3 Numerical source data.
Source Data Extended Data Fig. 5 Numerical source data.
Source Data Extended Data Fig. 6 Numerical source data.
Source Data Extended Data Fig. 7 Numerical source data.
Source Data Extended Data Fig. 8 Numerical source data.
