# Supplementary material for: Complement C1q-dependent excitatory and inhibitory synapse elimination by astrocytes and microglia in Alzheimer’s disease mouse models
Source: Nat Aging. 2022 Sep 20;2(9):837–50. doi: 10.1038/s43587-022-00281-1 (PMC10154216; doi:10.1038/s43587-022-00281-1)
Supplement: Supplementary file 1 — Reporting Summary. [file 43587_2022_281_MOESM1_ESM.pdf]

nature portfolio

Corresponding author(s): Borislav Dejanovic and Jesse Hanson

Last updated by author(s): Aug 2, 2022

Reporting Summary

Nature Portfolio wishes to improve the reproducibility of the work that we publish. This form provides structure for consistency and transparency in reporting. For further information on Nature Portfolio policies, see our Editorial Policies and the Editorial Policy Checklist.

Statistics

For all statistical analyses, confirm that the following items are present in the figure legend, table legend, main text, or Methods section.

n/a	Confirmed	
☐	☒	The exact sample size (*n*) for each experimental group/condition, given as a discrete number and unit of measurement
☐	☒	A statement on whether measurements were taken from distinct samples or whether the same sample was measured repeatedly
☐	☒	The statistical test(s) used AND whether they are one- or two-sided<br>*Only common tests should be described solely by name; describe more complex techniques in the Methods section.*
☒	☐	A description of all covariates tested
☐	☒	A description of any assumptions or corrections, such as tests of normality and adjustment for multiple comparisons
☐	☒	A full description of the statistical parameters including central tendency (e.g. means) or other basic estimates (e.g. regression coefficient) AND variation (e.g. standard deviation) or associated estimates of uncertainty (e.g. confidence intervals)
☐	☒	For null hypothesis testing, the test statistic (e.g. *F*, *t*, *r*) with confidence intervals, effect sizes, degrees of freedom and *P* value noted<br>*Give P values as exact values whenever suitable.*
☒	☐	For Bayesian analysis, information on the choice of priors and Markov chain Monte Carlo settings
☒	☐	For hierarchical and complex designs, identification of the appropriate level for tests and full reporting of outcomes
☐	☒	Estimates of effect sizes (e.g. Cohen's *d*, Pearson's *r*), indicating how they were calculated

Our web collection on statistics for biologists contains articles on many of the points above.

Software and code

Policy information about availability of computer code

Data collection	GSNAP: Version 2013-11-01, HT-Seq Genie: Version 4.4.2, Mascot: Version 2.4.1,
Data analysis	GraphPad Prism (v9), Imaris 9.6.1, MSstats v3.14.1, R: Version 4.0.0, Limma/Voom: Version 3.12, Fiji version 2.3.0

For manuscripts utilizing custom algorithms or software that are central to the research but not yet described in published literature, software must be made available to editors and reviewers. We strongly encourage code deposition in a community repository (e.g. GitHub). See the Nature Portfolio guidelines for submitting code & software for further information.

Data

Policy information about availability of data

All manuscripts must include a data availability statement. This statement should provide the following information, where applicable:

- Accession codes, unique identifiers, or web links for publicly available datasets
- A description of any restrictions on data availability
- For clinical datasets or third party data, please ensure that the statement adheres to our policy

All data are available from the authors upon request. The following datasets we have generated have been deposited to public repositories:
Single Cell RNAseq including P301S mice: GSE180041
Bulk RNAseq P301S x C1qKO: GSE186414
Proteomics data: ftp://massive.ucsd.edu/MSV000088313/
Other datasets we used for analysis are publicly available:
mouse reference genome (GRCm38): GCA_000001635.2

Field-specific reporting

Please select the one below that is the best fit for your research. If you are not sure, read the appropriate sections before making your selection.

☒ Life sciences ☐ Behavioural & social sciences ☐ Ecological, evolutionary & environmental sciences

For a reference copy of the document with all sections, see nature.com/documents/nr-reporting-summary-flat.pdf

Life sciences study design

All studies must disclose on these points even when the disclosure is negative.

Sample size	Sample sizes were determined based on previous experience (Dejanovic et al., Neuron 2018 and Wu, Dejanovic et al., Cell Reports 2019) for each experiment to yield high power to detect specific effects. No statistical methods were used to predetermine sample size.
Data exclusions	No data were excluded from the analyses
Replication	Experimental findings were replicated across orthogonal assays. For example, the result of the synaptic proteomics identifying association of astrocytes with synapses was reproduced in immunoEM experiments as well as IHC experiments
Randomization	Allocation was random.
Blinding	Data collection and statistical analyses were analyzed blinded to the experimental conditions.

Reporting for specific materials, systems and methods

We require information from authors about some types of materials, experimental systems and methods used in many studies. Here, indicate whether each material, system or method listed is relevant to your study. If you are not sure if a list item applies to your research, read the appropriate section before selecting a response.

Materials & experimental systems

n/a	Involved in the study	
☐	☒	Antibodies
☒	☐	Eukaryotic cell lines
☒	☐	Palaeontology and archaeology
☐	☒	Animals and other organisms
☐	☒	Human research participants
☒	☐	Clinical data
☒	☐	Dual use research of concern

Methods

n/a	Involved in the study	
☒	☐	ChIP-seq
☒	☐	Flow cytometry
☐	☒	MRI-based neuroimaging

Antibodies

Antibodies used	The following antibodies were used: anti-AT8 mouse monoclonal (ThermoScientific MN1020B, IHC 1:5,000), anti-GFAP rabbit polyclonal (Dako Z0334, IHC 1:20,000), anti-Iba1 rabbit monoclonal (Abcam ab178846, IHC 1:100,000), anti-NeuN mouse monoclonal (Millipore MAB377, IHC 1:1,500), anti-C1q rabbit monoclonal clone 4.8 (Abcam ab182451, IHC 1:1000); anti-GFAP mouse monoclonal (Thermo Fisher MA5-12023, IHC 1:1000), anti-Iba1 rabbit polyclonal (Wako 019-19741, IHC 1:1000), anti-LAMP1 rat monoclonal (Biolegend AB_572020, IHC 1:250), anti-Homer1 chicken polyclonal (Synaptic System 160006, IHC 1:1000); anti-Gephyrin guinea pig monoclonal (Synaptic System 147318, IHC 1:750); anti-C4 rabbit polyclonal (abx102219, Abbexa); anti-C4c, mouse monoclonal (A211, Quidel, San Diego, CA, USA); anti-C4c mouse monoclonal (C7850-18B1, US Biological); anti-C4 mouse monoclonal (LS-C128299, LSBio, Seattle, WA, USA); anti-FB mouse monoclonal (ab17927, Abcam) anti–Bb mouse monoclonal (Genentech PRO443354, clone 2F12); anti-Bb mouse monoclonal (A252, Quidel); anti-C3 rabbit polyclonal (A0063, Dako); anti-Eaat2/Glt1 guinea pig polyclonal (AB1783, Millipore).<br>Secondary antibodies: Goat anti-Mouse IgG (H+L) Highly Cross-Adsorbed Secondary Antibody, Alexa Fluor Plus 405 (ThermoFisher, A48225); Goat Anti-Rat IgG H&L (Alexa Fluor® 488) preadsorbed (Abcam, ab150165); Goat Anti-Chicken IgY H&L (Alexa Fluor® 555) preadsorbed (Abcam, ab150174); Goat anti-Guinea Pig IgG (H+L) Highly Cross-Adsorbed Secondary Antibody, Alexa Fluor 633 (ThermoFisher, A21105); Goat anti-Rabbit IgG (H+L) Highly Cross-Adsorbed Secondary Antibody, Alexa Fluor 680 (ThermoFisher, A21109); Goat anti-Rabbit IgG (H+L) Highly Cross-Adsorbed Secondary Antibody, Alexa Fluor 594 (ThermoFischer A11012)”
Validation	IHC:<br>-anti-AT8, mouse monoclonal (ThermoScientific MN1020B), Vendor (IHC, ELISA)<br>-anti-GFAP, rabbit polyclonal (Dako Z0334), Vendor (IHC)<br>anti-Iba1, rabbit monoclonal (Abcam ab178846), Vendor (IHC, WB, FC, ICC)<br>-anti-NeuN, (Millipore MAB377), Vendor (FC, IC, IF, IH, IP, WB)<br>IF:

-anti-C1q rabbit monoclonal (Abcam ab182451), KO validated Stephan AH et al. J Neurosci 2013 (IHC mouse); Vendor (IHC)
-anti-GFAP mouse monoclonal (Thermo Fisher MA5-12023), Vendor (WB, IHC, ICC)
-anti-Iba1 rabbit polyclonal (Wako 019-19741), Vendor (IHC, ICC)
-anti-LAMP1 rat monoclonal (Biolegend AB_572020, IHC 1:250), Vendor (FC, WB, ICC, IP)
-anti-Homer1 chicken polyclonal (Synaptic System 160006), Vendor (WB, ICC, IHC)
-anti-Gephyrin guinea pig monoclonal (Synaptic System 147318), KO validated, Vendor (ICC, IHC)
-anti-C3 rabbit polyclonal (Dako, A0063), Wilton et al. bioRxiv 2021

SIMOA:
-The antibody pairs used in C4 SIMOA measurements consisting of 1) anti-C4 (abx102219, Abbexa) for capture with anti-C4c (A211, Quidel, San Diego, CA, USA) for detection, and 2) anti-C4c (C7850-18B1, US Biological) for capture with anti-C4 (LS-C128299, LSBio, Seattle, WA, USA) for detection, were validated as specific for intact and processed C4, respectively, using purified human C4 proteins (CompTech).
-The antibody pairs used in FB SIMOA measurements consisting of 1) anti-FB (ab17927, Abcam) for capture and anti–Bb (Genentech) for detection, and 2) anti–Bb (Genentech) for capture with anti-Bb (A252, Quidel) for detection, were validated as specific for intact and processed FB, respectively, using purified human FB proteins (CompTech).

IEM:
- anti-Eaat2/Glt1 guinea pig polyclonal (AB1783, Millipore), Vendor IH(P), IF, WB

Animals and other organisms

Policy information about studies involving animals; ARRIVE guidelines recommended for reporting animal research

Laboratory animals	PS19 mice (Yoshiyama et al., 2007) were crossed to C1qC knockout mice (Jax stock #029409). 6 and 9 months old male mice were used for the study. Mice were group-housed up to 5 mice per cage in individually ventilated cages within animal rooms maintained on a 14:10-hour, light:dark cycle. Animal rooms were temperature and humidity-controlled, between 20-26°C and 30-70% respectively, with 10 to 15 room air exchanges per hour. Mice had ad libitum access to water and food. All testing occurred during the light phase.
Wild animals	No wild animals were used for this study.
Field-collected samples	This study did not involve samples collected from the field.
Ethics oversight	All animal studies were authorized and approved by the Genentech Institutional Animal Care and Use Committee.

Note that full information on the approval of the study protocol must also be provided in the manuscript.

Human research participants

Policy information about studies involving human research participants

Population characteristics	CSF biospecimens from Alzheimer's patients and healthy controls were obtained from Folio Biosciences using the same samples and patient population characteristics that were previously described (Wu et al Cell Reports 2019 and Wildsmith et al Mol. Neurodegener. 2014)
Recruitment	N/A
Ethics oversight	Folio Biosciences

Note that full information on the approval of the study protocol must also be provided in the manuscript.

Magnetic resonance imaging

Experimental design

Design type	N/A no clinical MRI was performed, MRI was only used to measure mouse brain volumes in this study
Design specifications	*Specify the number of blocks, trials or experimental units per session and/or subject, and specify the length of each trial or block (if trials are blocked) and interval between trials.*
Behavioral performance measures	*State number and/or type of variables recorded (e.g. correct button press, response time) and what statistics were used to establish that the subjects were performing the task as expected (e.g. mean, range, and/or standard deviation across subjects).*

Acquisition

Imaging type(s) | *Specify: functional, structural, diffusion, perfusion.*

Field strength | *Specify in Tesla*

Sequence & imaging parameters | *Specify the pulse sequence type (gradient echo, spin echo, etc.), imaging type (EPI, spiral, etc.), field of view, matrix size, slice thickness, orientation and TE/TR/flip angle.*

Area of acquisition | *State whether a whole brain scan was used OR define the area of acquisition, describing how the region was determined.*

Diffusion MRI ☐ Used ☐ Not used

Preprocessing

Preprocessing software | *Provide detail on software version and revision number and on specific parameters (model/functions, brain extraction, segmentation, smoothing kernel size, etc.).*

Normalization | *If data were normalized/standardized, describe the approach(es): specify linear or non-linear and define image types used for transformation OR indicate that data were not normalized and explain rationale for lack of normalization.*

Normalization template | *Describe the template used for normalization/transformation, specifying subject space or group standardized space (e.g. original Talairach, MNI305, ICBM152) OR indicate that the data were not normalized.*

Noise and artifact removal | *Describe your procedure(s) for artifact and structured noise removal, specifying motion parameters, tissue signals and physiological signals (heart rate, respiration).*

Volume censoring | *Define your software and/or method and criteria for volume censoring, and state the extent of such censoring.*

Statistical modeling & inference

Model type and settings | *Specify type (mass univariate, multivariate, RSA, predictive, etc.) and describe essential details of the model at the first and second levels (e.g. fixed, random or mixed effects; drift or auto-correlation).*

Effect(s) tested | *Define precise effect in terms of the task or stimulus conditions instead of psychological concepts and indicate whether ANOVA or factorial designs were used.*

Specify type of analysis: ☐ Whole brain ☐ ROI-based ☐ Both

Statistic type for inference (See Eklund et al. 2016) | *Specify voxel-wise or cluster-wise and report all relevant parameters for cluster-wise methods.*

Correction | *Describe the type of correction and how it is obtained for multiple comparisons (e.g. FWE, FDR, permutation or Monte Carlo).*

Models & analysis

n/a	Involved in the study
☐	☐ Functional and/or effective connectivity
☐	☐ Graph analysis
☐	☐ Multivariate modeling or predictive analysis

Functional and/or effective connectivity | *Report the measures of dependence used and the model details (e.g. Pearson correlation, partial correlation, mutual information).*

Graph analysis | *Report the dependent variable and connectivity measure, specifying weighted graph or binarized graph, subject- or group-level, and the global and/or node summaries used (e.g. clustering coefficient, efficiency, etc.).*

Multivariate modeling and predictive analysis | *Specify independent variables, features extraction and dimension reduction, model, training and evaluation metrics.*
